# Repositioning of the Angiotensin II Receptor Antagonist Candesartan as an Anti-Inflammatory Agent With NLRP3 Inflammasome Inhibitory Activity

**DOI:** 10.3389/fimmu.2022.870627

**Published:** 2022-05-20

**Authors:** Wen-Yu Lin, Lan-Hui Li, Ya-Yun Hsiao, Wei-Ting Wong, Hsiao-Wen Chiu, Hsien-Ta Hsu, Yi-Jen Peng, Chen-Lung Ho, Oleg V. Chernikov, Shu-Meng Cheng, Shih-Ping Yang, Kuo-Feng Hua

**Affiliations:** ^1^ Division of Cardiology, Department of Internal Medicine, Tri-Service General Hospital, National Defense Medical Center, Taipei, Taiwan; ^2^ Department of Laboratory Medicine, Linsen, Chinese Medicine and Kunming Branch, Taipei City Hospital, Taipei, Taiwan; ^3^ Department of Pathology, Tri-Service General Hospital, National Defense Medical Center, Taipei, Taiwan; ^4^ Department of Biotechnology and Animal Science, National Ilan University, Ilan, Taiwan; ^5^ Division of Neurosurgery, Taipei Tzu Chi Hospital, Buddhist Tzu Chi Medical Foundation, New Taipei City, Taiwan; ^6^ School of Medicine, Bu ddhist Tzu Chi University, Hualien, Taiwan; ^7^ Division of Wood Cellulose, Taiwan Forestry Research Institute, Taipei, Taiwan; ^8^ G.B. Elyakov Pacific Institute, Bioorganic Chemistry of the Far-Eastern Branch of the Russian Academy of Sciences (FEB RAS), Vladivostok, Russia; ^9^ Department of Medical Research, China Medical University Hospital, China Medical University, Taichung, Taiwan

**Keywords:** NLRP3 inflammasome, candesartan, angiotensin II receptor antagonist, drug repositioning, peritonitis

## Abstract

Aberrant activation of the NLRP3 inflammasome promotes the pathogenesis of many inflammatory diseases. The development of the NLRP3 inflammasome inhibitors from existing drugs for new therapeutic purposes is becoming more important. Candesartan is an angiotensin II receptor antagonist widely used as a blood pressure-lowering drug; however, the inhibitory potential of candesartan on the NLRP3 inflammasome has not yet been investigated. We demonstrated that candesartan significantly inhibited the NLRP3 inflammasome and pyroptosis in macrophages. Mechanistic analysis revealed that candesartan inhibited the expression of NLRP3 and proIL-1β by suppressing NF-κB activation and reducing the phosphorylation of ERK1/2 and JNK1/2. Candesartan reduced mitochondrial damage and inhibited the NLRP3 inflammasome assembly by suppressing NLRP3 binding to PKR, NEK7 and ASC. In addition, candesartan inhibited IL-1β secretion partially through autophagy induction. Furthermore, oral administration of candesartan reduced peritoneal neutrophil influx, NLRP3 and ASC expression in peritoneal cells, and lavage fluid concentrations of active caspase-1, IL-1β, IL-6 and MCP-1 in uric acid crystal-injected mice. These results indicated that candesartan has board anti-inflammatory effects and has the potential to be repositioned to ameliorate inflammatory diseases or NLRP3-associated complications.

## Introduction

Pathogenic inflammation is traditionally caused by pathogen infection, leading to the production of proinflammatory mediators, such as TNF-α, IL-6 and nitric oxide. Whereas infection is the major event causing inflammation, host-derived or environmental danger signals have been demonstrated to induce sterile inflammation (1). Host-derived danger signals such as saturated fatty acids and islet amyloid polypeptide cause type II diabetes, cholesterol crystals cause atherosclerosis, uric acid crystals cause gout inflammation, and amyloid-β causes Alzheimer’s disease (2). Environmental danger signals, such as PM2.5 in air pollution and nanoparticles in the electronics industry, cause airway and pulmonary inflammation (3, 4). Inflammsomes are cytosolic protein complexes that recognize and respond to pathogen infection and diverse sterile danger signals to promote the development of inflammatory disease (5). Among the identified inflammasomes, the NLRP3 inflammasome not only responds to pathogen infections but is also triggered by sterile host-derived or environmental danger signals (6). Although the NLRP3 inflammasome is important for host defense against various infections and has a beneficial role in some cancers, it responds to a broad range of medically relevant stimuli and promotes the pathogenesis of inflammation-associated diseases such as cardiovascular disease, diabetes and obesity (7). Therefore, targeting the NLRP3 inflammasome has become a highly desirable drug target to treat a wide range of human diseases (8).

Small molecule inhibitors targeting the NLRP3 inflammasome offer a new therapeutic strategy in new drug development (8). MCC950, a small-molecule inhibitor of NLRP3, directly binds the NLRP3 ATP hydrolysis motif and closes the active conformation of NLRP3 for NLRP3 inflammasome inhibition, showing therapeutic potential for NLRP3-associated disorders (9–11). However, MCC950 did not yet hit the market, as some side effects were observed. Although several NLRP3-specific inhibitors are in clinical practice or in development, for examples, Dapansutrile from Olatec (phase II), Somalix from Inflazome (phase II), IFM-2427 from IFM Tre/Novartis (phase I), NT-0167 from Nodthera (phase I), and a none disclosed inhibitor from Jecure/Genentech (preclinical), the toxic risk, high cost and high failure rate make it difficult to achieve (12). In recent years, drug repositioning has attracted particular attention from researchers and pharmaceutical industries due to its pharmacokinetic and safety profile, which makes it cost and time effective.

We have identified the β-blocker carvedilol as a novel autophagy inducer that inhibits the NLRP3 inflammasome in macrophages and ameliorates NLRP3-associated peritonitis in mice (13). Candesartan (CS), an angiotensin II type 1 receptor blocker, is widely used as the first-line drug treatment for hypertension. An increasing number of studies have indicated that CS has additional beneficial effects in diabetes, stroke, dementia and atrial fibrillation, suggesting the repositioning potential of CS (14). However, the effect of CS on NLRP3 inflammasome activity has never been investigated. In this study, we investigated the effect of CS on NLRP3 inflammasome inhibitory activity of CS *in vitro* and *in vivo*.

## Materials and Methods

### Materials

Candesartan (sc-217825A), Irbesartan (sc-218603), phorbol 12-myristate 13-acetate (sc-3576), CRISPR/Cas9 knockout plasmids targeting LC3 (sc-426563 and sc-417828-HDR) and antibodies against actin (sc-47778) and ASC (sc-25514-R) were purchased from Santa Cruz Biotechnology (Santa Cruz, CA). ATP (tlrl-atpl), nigericin (tlrl-nig), monosodium urate (MSU) (tlrl-msu), Pam3CSK4 (tlrl-pms), poly(dA/dT) (tlrl-patn), muramyl dipeptide (MDP) (tlrl-mdp), flagellin from *Salmonella typhimurium* (FLA-ST) (tlrl-stfla), NF-κB t reporter gene pNiFty2-SEAP plasmids (pnifty2-seap) and QUANTI-Blue medium (rep-qb2) were purchased from *In vivo*Gen (San Diego, CA). LPS (L2630), monodansylcadaverine (MDC) (D4008), acridine orange (AO) (A9231) and Histopaque-1077 (10771-100ML) were purchased from Sigma–Aldrich (St. Louis, MO). Antibodies against IL-1β (AB-401-NA) were purchased from R&D Systems (Minneapolis, MN). Antibodies against phospho-MAPK (#9910) were purchased from Cell Signaling Technology (Beverly, MA). Antibodies against caspase-1 (AG-20B-0044) and NLRP3 (AG-20B-0014) were purchased from Adipogen Life Science (San Diego, CA). Antibodies against IL-18 (ab71495), NIMA-related kinase 7 (NEK7) (ab96538), double-stranded RNA-dependent protein kinase (PKR) (ab184257) and phospho-PKR (ab32036) were purchased from Abcam (Cambridge, UK). Antibodies against caspase-11 (NB120-10454SS), GSDMDC1 (NBP2-33422), LC3B (NB100-2220) and p62 (#5114) were purchased from Novus Biologicals (Littleton, CO). ELISA kits for IL-1β (88-7013-88), IL-6 (88-7064-88) and MCP-1 (88-7391-88), antibodies against Gr1 (12-5931-82) and CD45 (11-0459-42), MitoTracker Deep Red (M22426), MitoTracker Green (M7514), DiOC_2_(3) (M34150), MitoSOX (M36008), CM-H_2_DCFDA (C6827), disuccinimidyl suberate (21655), AlamarBlue cell viability reagent (DAL1025), LDH cytotoxicity assay kit (C20300), RPMI-1640 medium and fetal bovine serum were purchased from Thermo Fisher Scientific (Waltham, MA). An ELISA kit for active caspase-1 (IB99502) was purchased from IBL-America (Minneapolis, MN).

### Cell Culture

The J774A.1 mouse macrophage cell line was purchased from the American Type Culture Collection (Rockville, MD). Human THP-1 macrophages were differentiated from human THP-1 monocytes by incubation for 48 h with 50 nM phorbol 12-myristate 13-acetate. Human primary peripheral blood mononuclear cells (PBMC) were isolated from whole blood from healthy volunteers. Briefly, free collected whole blood was separated by Histopaque-1077 using density gradient centrifugation. The whole blood collection was performed in accordance with the guidelines and regulations provided and accepted by the Institutional Review Board of the Tri-Service General Hospital, National Defense Medical Center (TSGH-IRB-2-106-05-190 and TSGH-IRB-2-106-05-009). NF-κB transcriptional activity reporter cells (J-Blue cells) were established by J774A.1 macrophages stably transfected with the pNiFty2-SEAP plasmids and selected with medium containing100 μg/ml Zeocin. Generation of LC3-knockout macrophages, J774A.1 macrophages were transfected with CRISPR/Cas9 knockout plasmids targeting LC3 and the cells were selected with medium containing 5 μg/ml puromycin. All cells were propagated in RPMI-1640 medium containing 10% heat-inactivated fetal bovine serum at 37°C in a 5% CO_2_ incubator.

### Activation of the Inflammasomes

For the NLRP3 inflammasome activation, 2×10^5^ J774A.1 macrophages, THP-1 macrophages or PBMC were plated per well in 0.5 ml of culture medium overnight and then primed with 1 μg/ml LPS for 5 h. The cells were then incubated with CS for 0.5 h followed by treatment with 10 μM nigericin or 5 mM ATP for 0.5 h. For the NLRC4 inflammasome activation, 2×10^5^ J774A.1 macrophages were plated per well in 0.5 ml of culture medium overnight and then primed with 1 μg/ml LPS for 5 h. The cells were then incubated with CS for 0.5 h followed by *Salmonella* (ATCC 14028) infection at a multiplicity of infection (MOI) of 20 for 2 h or transfection with 1 μg/ml FLA-ST for 6 h. For the AIM2, non-canonical and NLRP1 inflammasome activation, 2×10^5^ J774A.1 macrophages were plated per well in 0.5 ml of culture medium overnight and then primed with 1 μg/ml LPS or 1 μg/ml Pam3CSK4 (for non-canonical inflammasome) for 5 h. The cells were then incubated with CS for 0.5 h followed by transfection with 2 μg/ml poly(dA/dT), 2 μg/ml LPS or 10 μg/ml MDP for 6 h. The IL-1β levels in the supernatants were analysed by ELISA. For the detection of proIL-1β/IL-1β, IL-18, caspase-1 (p10 and p45), NLRP3 and ASC in the supernatants, 2×10^6^ J774A.1 macrophages per well were plated in 2 ml of culture medium overnight and then primed with 1 μg/ml LPS for 5 h in serum-free medium. The cells were then incubated with CS for 0.5 h followed by treatment with 10 μM nigericin or 5 mM ATP for an additional 0.5 h or *Salmonella* infection at a MOI of 20 for 2 h. The supernatants were concentrated as described in our previous study, and the protein expression levels were analysed by Western blot (15). For the effect of CS on LPS-induced expression of proIL-1β and NLRP3, 2×10^6^ J774A.1 macrophages per well were plated in 2 ml of culture medium overnight and then incubated with CS for 0.5 h followed by 1 μg/ml LPS treatment for 6 h. The expression levels of proIL-1β and NLRP3 in the cell lysates were analysed by Western blot. The intensity of the Western blot images was analysed by ImageJ.

### Pyroptosis Assay

For the caspase-11 activation and GSDMD cleavage, 2×10^5^ J774A.1 macrophages were plated per well in 0.5 ml of culture medium overnight and then primed with 1 μg/ml LPS for 5 h. The cells were then incubated with CS for 0.5 h followed by treatment with 10 μM nigericin for 0.5 h. The levels of pro-caspase-11/active caspase-11 and full length GSDMD/C-terminal GSDMD in the supernatants were analysed by Western blot. The intensity of the Western blot images was analysed by ImageJ. For the cell viability assay, 2×10^3^ J774A.1 macrophages were plated per well in 0.1 ml of culture medium overnight and then primed with 1 μg/ml LPS for 5 h followed by incubation with or without CS for 0.5 h. The cells were then incubated with 10 μM nigericin for 0.5 h. Cell viability was analysed by alamarBlue cell viability reagent according to the manufacturer’s instructions. For the LDH release assay, 2×10^5^ J774A.1 macrophages were plated per well in 0.5 ml of culture medium overnight and then primed with 1 μg/ml LPS for 5 h followed by incubation with or without CS for 0.5 h. The cells were then incubated with 10 μM nigericin for 0.5 h. The levels of LDH in the supernatants were analysed by an LDH cytotoxicity assay kit according to the manufacturer’s instructions.

### NF-κB Transcriptional Assay

2×10^5^ J-Blue cells were plated per well in 0.5 ml of culture medium overnight and then incubated with CS for 0.5 h, followed by treatment with 1 μg/ml LPS for 24 h. The NF-κB transcriptional activity was analysed using QUANTI-Blue as described previously (16).

### Mitochondrial Damage Assay

2×10^6^ J774A.1 macrophages were plated per well in 2 ml of culture medium overnight and then primed with 1 μg/ml LPS for 5 h followed by incubation with or without CS for 0.5 h. Cells were then incubated with 10 μM nigericin for 0.5 h. To analyse mitochondrial integrity, cells were stained with 25 nM intact mitochondrial dye MitoTracker Deep Red and 25 nM total mitochondrial dye MitoTracker Green for 15 min. To analyse mitochondrial ROS, cells were stained with 5 μM mitochondrial ROS dye MitoSOX for 15 min. The fluorescent signals were acquired by flow cytometry.

### Detection of Intracellular ROS

2×10^6^ J774A.1 macrophages were plated per well in 2 ml of culture medium overnight and then incubated with or without 15 μM CS for 0.5 h followed by staining with 2 μM CM-H_2_DCFDA for 15 min. Cells were then incubated with 1 μg/ml LPS for 30 min, and the intracellular fluorescence intensity was detected by flow cytometry.

### Mouse Model of MSU-Induced Peritonitis

C57BL/6JNal mice (male, 8 weeks old) were purchased from The National Laboratory Animal Centre (Taipei, Taiwan). The mice were housed in the animal centre of National Ilan University in a standard and controlled environment. The studies were performed with the approval and regulation of the Institutional Animal Care and Use Committee of the National Ilan University (No. 107-3). The mice were randomly divided into five groups: Control group (vehicle/vehicle): oral administration of 0.2 ml 0.5% DMSO in sterile PBS (vehicle #1) at 0, 24 and 48 h; i.p. injection of 0.5 ml sterile PBS (vehicle #2) at 1 and 49 h, n=3. MSU group **(**vehicle/MSU**)**: oral administration of vehicle #1 at 0, 24 and 48 h; i.p. injection of 3 mg sterile MSU crystals in 0.5 ml PBS at 1 and 49 h, n=6. CS+MSU group (CS/MSU): oral administration of 30 mg/kg CS at 0, 24 and 48 h; i.p. injection of 3 mg sterile MSU crystals in 0.5 ml PBS at 1 and 49 h, n=6. Colchicine+MSU group (Colchicine/MSU): i.p. injection of 1 mg/kg body weight colchicine at 48 h; i.p. injection of 3 mg sterile MSU crystals in 0.5 ml PBS at 1 and 49 h, n=5. CS group (CS/vehicle): oral administration of 30 mg/kg CS at 0, 24 and 48 h; i.p. injection of vehicle #2 at 1 and 49 h, n=3. Mice were euthanized at 53 h, and the peritonea were lavaged with 3 ml ice-cold PBS. The neutrophil peritoneal influx was quantified by Gr-1 and CD45 staining and analysed by flow cytometry. The expression levels of cytokines in peritoneal lavage fluids were measured by ELISA. The levels of NLRP3 and ASC in the peritoneal cells were measured by Western blot.

## Results

### CS Inhibits NLRP3 Inflammasome Activation

**Figure 1:**
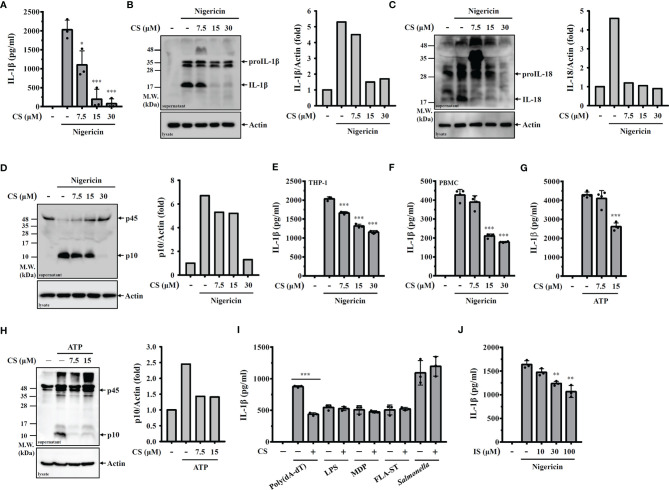
CS inhibits NLRP3 inflammasome activation. **(A–D)** J774A.1 macrophages were primed with LPS for 5 h and incubated with CS for 0.5 h Cells were stimulated with 10 μM nigericin for 0.5 h The levels of IL-1β in the supernatants were measured by ELISA **(A)** and Western blot **(B)**. The levels of IL-18 **(C)** and caspase-1 **(D)** in the supernatants were measured by Western blot. **(E, F)** Human THP-1 macrophages or PBMC were primed with LPS for 5 h and incubated with CS for 0.5 h Cells were stimulated with 10 μM nigericin for 0.5 h The levels of IL-1β in the supernatants were measured by ELISA. **(G, H)** J774A.1 macrophages were primed with LPS for 5 h and incubated with CS for 0.5 h Cells were stimulated with 5 mM ATP for 0.5 h The levels of IL-1β in the supernatants were measured by ELISA **(G)** and the levels of caspase-1 in the supernatants were measured by Western blot **(H)**. **(I)** J774A.1 macrophages were primed with LPS or Pam3CSK4 (for non-canonical inflammasome) for 5 h and incubated with CS for 0.5 h Cells were transfected with poly(dA/dT), LPS, MDP or FLA-ST for 6 h or infected with *Salmonella* for 2 h The levels of IL-1β in the supernatants were measured by ELISA. **(J)** J774A.1 macrophages were primed with LPS for 5 h and incubated with Irbesartan (IS) for 0.5 h Cells were stimulated with 10 μM nigericin for 0.5 h The levels of IL-1β in the supernatants were measured by ELISA. The ELISA data are expressed as the means ± SD of the three separate experiments. The Western blot images are representative results, and the histogram shows the band intensity. *, ** and *** indicate a significant difference at the level of *p*<0.05, *p*<0.01 and *p*<0.001, respectively, compared to nigericin- or ATP-activated cells or as indicated.

To investigate the inhibition potential of candesartan on NLRP3 inflammasome in macrophages, cells were primed with LPS for 5 h followed by incubation with CS (7.5, 15, 30 µM) for 0.5 h. The cells were then stimulated with the NLRP3 activator nigericin for an additional 0.5 h. We found that CS dose-dependently inhibited IL-1β secretion, as analysed by ELISA (**Figure 1A**) and Western blot (**Figure 1B**). The NLRP3 inflammasome inhibitory effect of CS was confirmed by reduced IL-18 secretion (**Figure 1C**) and active caspase-1 (p10) expression (**Figure 1D**) analysed by Western blot. CS also inhibited IL-1β secretion in nigericin-activated human THP-1 macrophages (**Figure 1E**) and PBMC (**Figure 1F**). In addition, to investigate whether the NLRP3 inflammasome inhibitory effect of CS is specific to nigericin-activated cells, we investigated the effect of CS on IL-1β secretion in macrophages in response to other NLRP3 activator. We found that CS also inhibited the IL-1β secretion (**Figure 1G**) and caspase-1 activation (**Figure 1H**) induced by the NLRP3 activator ATP. Furthermore, we investigate whether CS specifically inhibits the NLRP3 inflammasome or also affects other inflammasomes. We found that CS was not selective for NLRP3-dependent inflammasome stimuli, as CS also reduced IL-1β secretion in J774A.1 macrophages transfected with poly(dA:dT), which is dependent on the AIM2 inflammasome (**Figure 1I**). However, CS did not reduce IL-1β secretion in J774A.1 macrophages transfected with LPS, MDP, FLA-ST, which are dependent on the non-canonical-, NLRP1- and NLRC4-inflammasome, respectively (**Figure 1I**). The effect of CS on the NLRC4 inflammasome was confirmed, as it did not affect the IL-1β secretion in J774A.1 macrophages infected with *Salmonella* (**Figure 1I**). We further investigate whether the inhibitory effect of CS on the NLRP3 inflammasome is through angiotensin II receptor inhibition or through the off-target effect. We tested the effect of another angiotensin II receptor blocker Irbesartan on the NLRP3 inflammasome activation, and found that Irbesartan inhibited IL-1β secretion in nigericin-activated J774A.1 macrophages, suggested that CS-mediated inhibition of the NLRP3 inflammasome may through angiotensin II receptor inhibition (**Figure 1J**).

### CS Inhibits Pyroptosis

**Figure 2:**
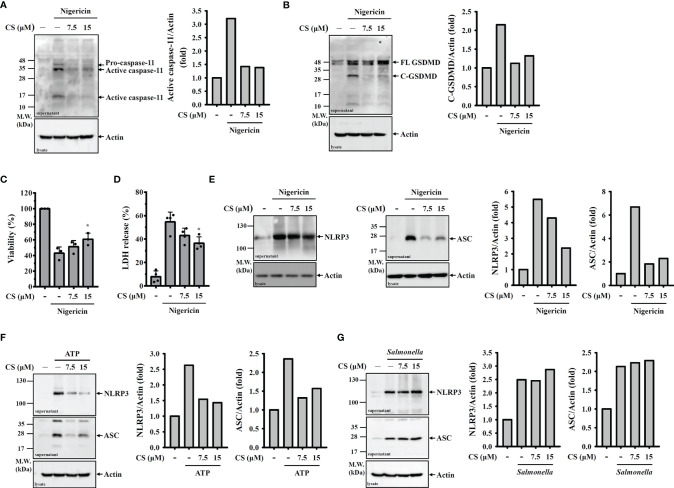
CS inhibits pyroptosis. **(A–E)** J774A.1 macrophages were primed with LPS for 5 h and incubated with CS for 0.5 h Cells were stimulated with 10 μM nigericin for 0.5 h The levels of caspase-11 **(A)** and GSDMD **(B)** in the supernatants were measured by Western blot. Cell viability was measured by the AlamarBlue assay **(C)**, and the levels of LDH in the supernatants were measured by the LDH release assay **(D)**. The levels of NLRP3 and ASC in the supernatants were measured by Western blot **(E)**. **(F, G)** J774A.1 macrophages were primed with LPS for 5 h and incubated with CS for 0.5 h Cells were stimulated with 5 mM ATP for 0.5 h **(F)** or infected with *Salmonella* for 2 h **(G)**. The levels of NLRP3 and ASC in the supernatants were measured by Western blot. The cell viability and LDH release data are expressed as the means ± SD of the three separate experiments. The Western blot images are representative results, and the histogram shows the band intensity. * indicates a significant difference at the level of *p*<0.05 compared to nigericin-activated cells.

Activation of caspase-11 or caspase-1 leads to the GSDMD cleavage and results in the plasma membrane pore formation and pyroptotic cell death, which is characterized by a loss of cell membrane integrity that leads to intracellular component release (17). We found that nigericin induced caspase-11 activation (**Figure 2A**) and GSDMD cleavage (**Figure 2B**) in nigericin-activated J774A.1 macrophages, and these effects were inhibited by CS. In addition, we found that CS reduced nigericin-mediated cell death (**Figure 2C**). Furthermore, we found that CS reduced nigericin-induced LDH release, indicating that the loss of membrane integrity was alleviated by CS (**Figure 2D**). CS also reduced NLRP3 and ASC release in nigericin- activated (**Figure 2E**) or ATP-activated (**Figure 2F**) J774A.1 macrophages, confirming the preservation effects of CS on cell membrane integrity. However, CS did not reduce the release of NLRP3 and ASC in *Salmonella*-infected macrophages (**Figure 2G**), which is consistent with the finding that candesartan did not reduce IL-1β secretion in J774A.1 macrophages infected with *Salmonella* (**Figure 1I**).

### CS Inhibits NLRP3 and proIL-1β Expression by Reducing NF-κB Activation and MAPK Phosphorylation

**Figure 3:**
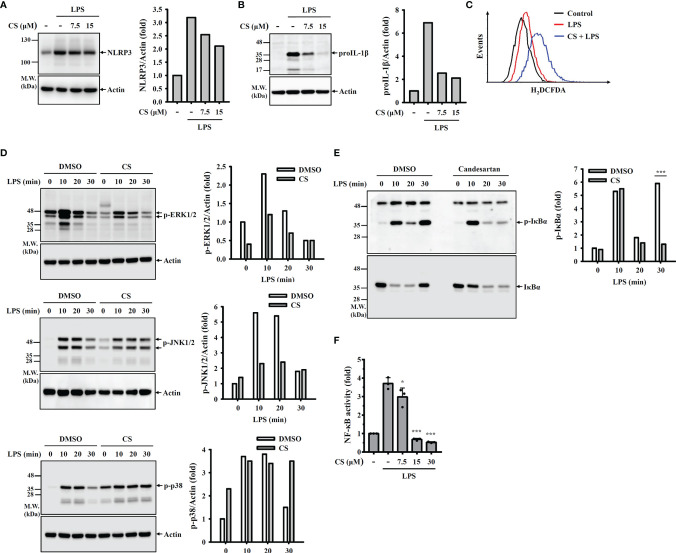
CS inhibits NLRP3 and proIL-1β expression by reducing NF-κB activation and MAPK phosphorylation. **(A, B)** J774A.1 macrophages were incubated with CS for 0.5 h followed by stimulation with LPS for 6 h The levels of NLRP3 **(A)** and proIL-1β **(B)** in the cell lysates were measured by Western blot. **(C)** J774A.1 macrophages were incubated with CS for 0.5 h followed by stimulation with LPS for 0.5 h Intracellular ROS were measured by H_2_DCFDA staining. **(D, E)** J774A.1 macrophages were incubated with 15 µM CS for 0.5 h followed by stimulation with LPS for 0-30 min. The phosphorylation levels of ERK1/2, JNK1/2, p38 **(D)**, or IκBα **(E)** in the cell lysates were measured by Western blot. **(F)** J-Blue cells were incubated with CS for 0.5 h followed by stimulation with LPS for 24 h NF-κB transcriptional activity was measured by QUANTI-Blue. The Western blot images are representative results, and the histogram shows the band intensity. The NF-κB transcriptional activity data are expressed as the means ± SD of the three separate experiments. * and *** indicate a significant difference at the level of *p*<0.05 and *p*<0.001, respectively, compared to LPS-activated cells.

Transcriptional upregulation of NLRP3 and proIL-1β by pathogen-associated molecular patterns through Toll-like receptor activation is the priming step of the NLRP3 inflammasome. We asked whether CS affects the priming step of the NLRP3 inflammasome. We found that CS slightly reduced NLRP3 expression (**Figure 3A**) but significantly inhibited proIL-1β expression (**Figure 3B**) in LPS-activated macrophages. Ligation of toll-like receptor 4 and LPS triggers ROS production, MAPK phosphorylation and NF-κB activation, which regulate NLRP3 and proIL-1β expression (18–20). Therefore, we examined whether CS reduced NLRP3 and proIL-1β expression by affecting these signalling events. We found that CS did not reduce but increased ROS production in LPS-activated macrophages (**Figure 3C**). In addition, the phosphorylation levels of ERK1/2 and JNK1/2 in LPS-activated macrophages were reduced by CS; however, p38 phosphorylation was not affected (**Figure 3D**). Furthermore, LPS-mediated IκBα phosphorylation (**Figure 3E**) and the transcriptional activity of NF-κB (**Figure 3F**) were significantly reduced by CS.

### CS Inhibits the Activation Signal by Reducing Mitochondrial Dysfunction

**Figure 4:**
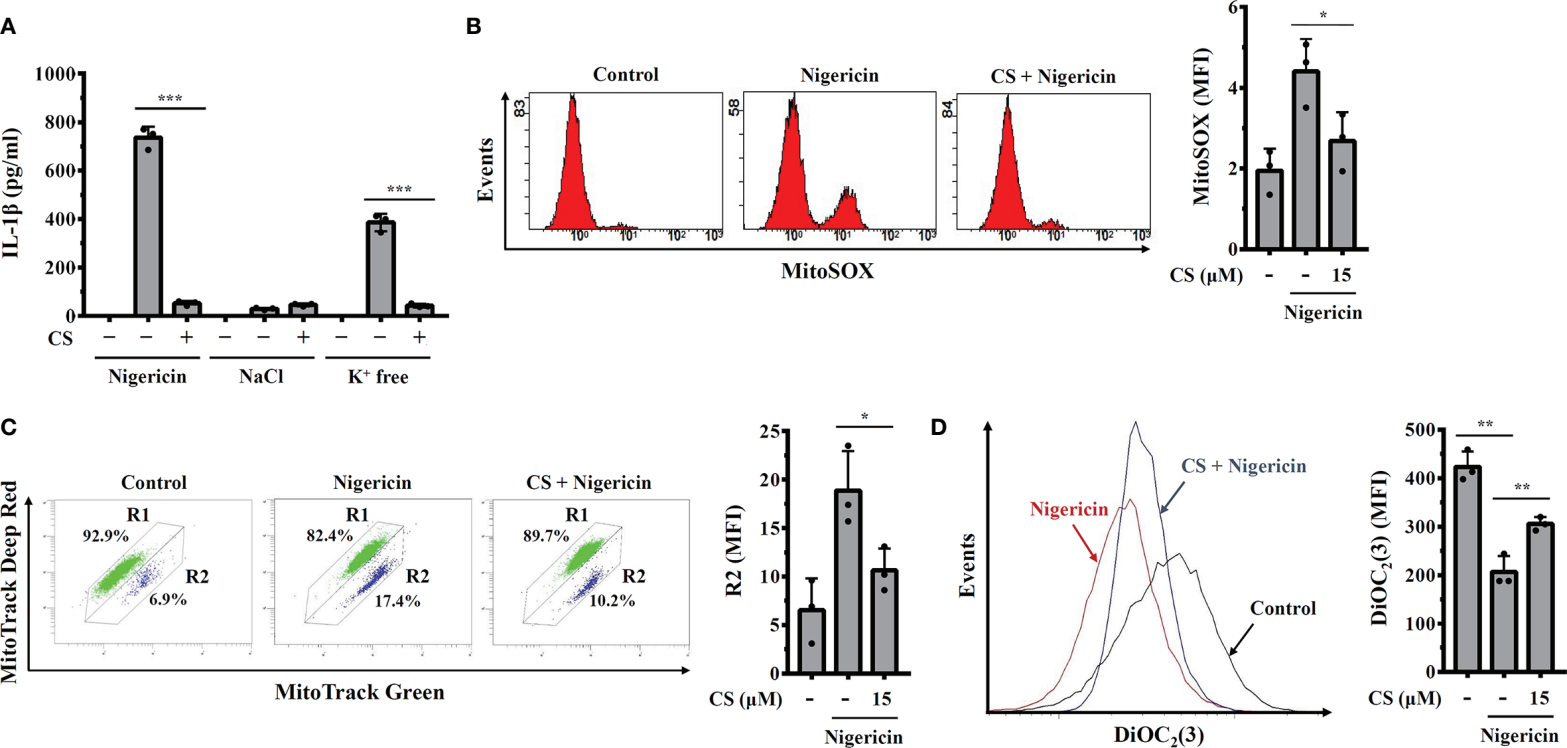
Candesartan reduces mitochondrial dysfunction. **(A)** J774A.1 macrophages were primed with LPS for 5 h and incubated with CS for 0.5 h Cells were stimulated with 10 μM nigericin for 0.5 h or incubated with NaCl or K^+^-free medium for 2 h The levels of IL-1β in the supernatants were measured by ELISA. **(B–D)** J774A.1 macrophages were primed with LPS for 5 h and incubated with CS for 0.5 h Cells were stimulated with 10 μM nigericin for 0.5 h Mitochondrial ROS were measured by MitoSOX staining **(B)**, mitochondrial integrity was measured by MitoTracker staining **(C)**, and mitochondrial membrane potential was measured by DiOC_2_(3) staining **(D)**. The ELISA data are expressed as the means ± SD of the three separate experiments. The mitochondrial data are representative results, and the histogram shows the quantitative results as the mean ± SD. *, ** and *** indicate a significant difference at the level of *p*<0.05, *p*<0.01 and *p*<0.001, respectively, as indicated.

NLRP3 activator-induced K^+^ efflux activates downstream signalling pathways leading to mitochondrial damage and NLRP3 inflammasome activation (21, 22). We asked whether CS inhibited K^+^ efflux induced NLRP3 inflammasome activation. We induced NLRP3 activator-independent K^+^ efflux by replacing the culture medium with K^+^-free medium or NaCl (as a control) in LPS-primed macrophages, which induces IL-1β secretion (15). We found that physically induced K^+^ efflux by K^+^-free medium increased IL-1β secretion in LPS-primed macrophages, and this effect was inhibited by CS (**Figure 4A**). These results indicated that CS inhibited the NLRP3 inflammasome by targeting the downstream events of K^+^ efflux. We further investigated whether CS inhibited mitochondrial dysfunction, a downstream event of K^+^ efflux that induces NLRP3 inflammasome activation (21). Using the mitochondrial ROS-specific indicator MitoSOX staining, we found that CS inhibited nigericin-mediated mitochondrial ROS production (**Figure 4B**). In addition, using the mitochondrial membrane integrity indicator MitoTracker Deep Red staining, we found that CS prevented nigericin-mediated mitochondrial membrane integrity loss (**Figure 4C**). The protective effect of CS on mitochondrial membrane integrity was confirmed by DiOC_2_ (3) staining, a membrane-potential-sensitive fluorescent dye that binds to intact mitochondrial membranes, as CS increased DiOC_2_ (3) staining in nigericin-treated cells (**Figure 4D**). These results demonstrated that CS inhibited mitochondrial dysfunction in NLRP3 inflammasome-activated macrophages.

### CS Inhibits NLRP3 Inflammasome Assembly

**Figure 5:**
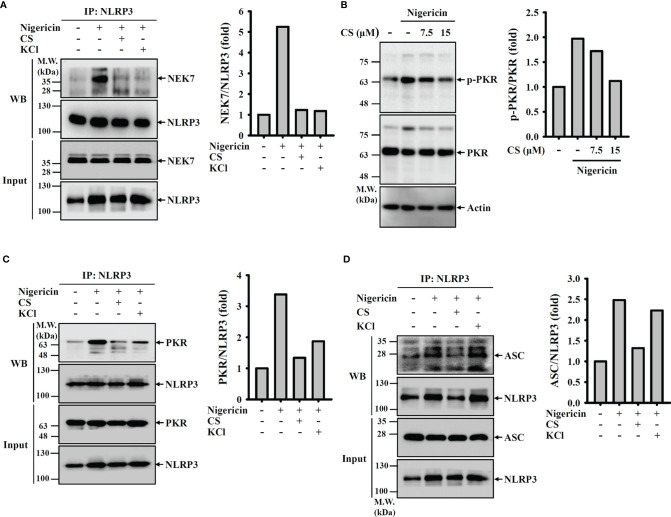
Candesartan inhibits NLRP3 inflammasome assembly. **(A, C, D)** J774A.1 macrophages were primed with LPS for 5 h and incubated with 15 μM CS or 50 mM KCl for 0.5 h Cells were stimulated with 10 μM nigericin for 0.5 h NLRP3 was immunoprecipitated from cell lysates and Western blot against NEK7 **(A)**, PKR **(C)** and ASC **(D)**. **(B)** J774A.1 macrophages were primed with LPS for 5 h and incubated with 15 μM CS for 0.5 h. Cells were stimulated with 10 μM nigericin for 0.5 h. The phosphorylation levels of PKR in the cell lysates were measured by Western blot. The Western blot images are representative results, and the histogram shows the band intensity.

We asked whether CS inhibited the NLRP3 inflammasome by preventing complex assembly. We found that NEK7, a protein that is essential for the assembly and activation of the NLRP3 inflammasome (23, 24), physically interacted with NLRP3 in nigericin-activated cells, and the interaction was inhibited by CS or the K^+^ efflux inhibitor KCl (**Figure 5A**). In addition, we investigated whether CS affects the phosphorylation level of another NLRP3-binding protein, PKR (25), and the interaction between PKR and NLRP3. We found that nigericin-mediated PKR phosphorylation was reduced by CS (**Figure 5B**). Similar to the K^+^ efflux inhibitor KCl, CS disrupted the binding between PKR and NLRP3 (**Figure 5C**). Furthermore, nigericin-mediated ASC/NLRP3 complex formation was reduced by CS and KCl (**Figure 5D**). These results indicated that CS suppressed NLRP3 inflammasome assembly.

### CS Inhibits the NLRP3 Inflammasome Partially Through Autophagy Induction

**Figure 6:**
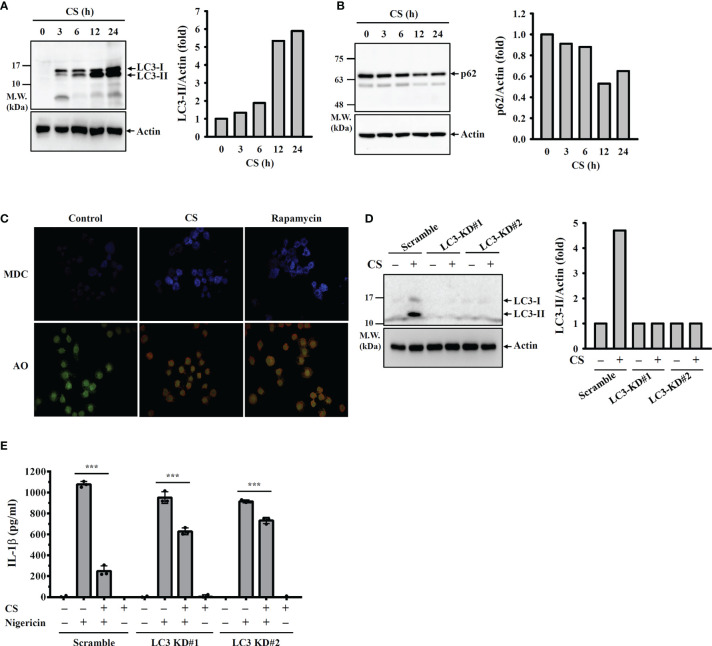
CS inhibits the NLRP3 inflammasome partially through autophagy induction. **(A, B)** J774A.1 macrophages were incubated with CS for 0-24 h The levels of LC3 **(A)** and p62 **(B)** in the cell lysates were measured by Western blot. **(C)** J774A.1 macrophages were incubated with 15 μM CS for 6 h or 100 nM rapamycin for 4 h The autophagic response was measured by MDC and AO staining. **(D)** Scramble control or LC3 knockdown J774A.1 macrophages were incubated with 15 μM CS for 24 h The levels of LC3 in the cell lysates were measured by Western blot. **(E)** Scramble control or LC3 knockdown J774A.1 macrophages were primed with LPS for 5 h and incubated with CS for 6 h Cells were stimulated with 10 μM nigericin for 0.5 h. The levels of IL-1β in the supernatants were measured by ELISA. The ELISA data are expressed as the means ± SD of the three separate experiments. The Western blot images are representative results, and the histogram shows the band intensity. *** indicates a significant difference at the level of *p*<0.001 as indicated.

Autophagy is a cellular protective reaction that limits the activation of the NLRP3 inflammasome (26). We found that CS induced autophagy in macrophages increased LC-3 expression (**Figure 6A**) and reduced p62 expression (**Figure 6B**), which are the characteristic features of autophagy. The autophagy induction activity of CS was confirmed, as it increased the fluorescent signals of the autophagolysosome marker MDC and the acidic organelle marker AO (**Figure 6C**). To investigate whether CS inhibits the NLRP3 inflammasome through autophagy induction, we knocked down LC-3 expression in macrophages by CRISPR/Cas9 targeting LC-3 (**Figure 6D**). In scramble control cells, CS significantly reduced IL-1β expression in nigericin-activated macrophages; however, the IL-1β inhibitory activity of CS was reduced in LC-3 knockdown cells (**Figure 6E**). These results indicated that CS inhibited the NLRP3 inflammasome partially through autophagy induction.

### Oral Administration of CS Reduces the NLRP3 Inflammasome in a Mouse Model of MSU-Induced Peritonitis

**Figure 7:**
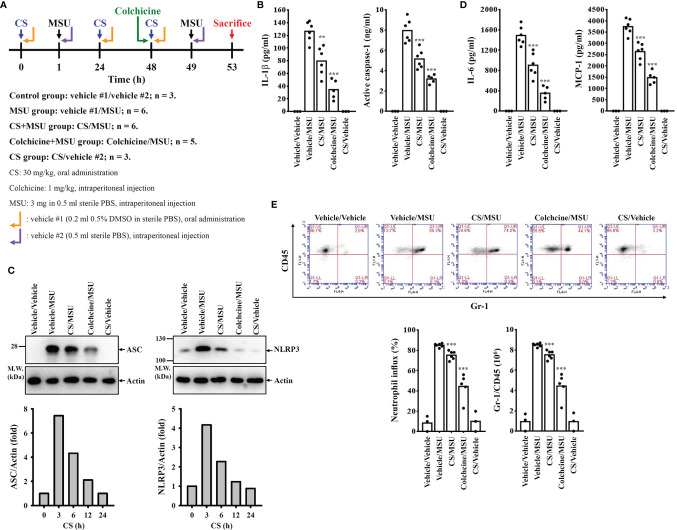
Oral administration of candesartan reduces the NLRP3 inflammasome activity in a mouse model of MSU-mediated peritonitis. **(A)** The scheme of the mice experiments. **(B)** The levels of IL-1β and active caspase-1 in the peritoneal lavage fluids were measured by ELISA. **(C)** The levels of ASC and NLRP3 in the peritoneal cells were measured by Western blot. **(D)** The levels of IL-6 and MCP-1 in the peritoneal lavage fluids were measured by ELISA. **(E)** Neutrophil influx was quantified by Gr-1 and CD45 staining. The Western blot images are representative results, and the histogram shows the band intensity. ** and *** indicate a significant difference at the level of *p*<0.01 and *p*<0.001, respectively, compared to the Vehicle/MSU group.

To investigate the in vivo NLRP3 inflammasome inhibition potential of CS, a mouse model of MSU-induced peritonitis with aberrant activation of the NLRP3 inflammasome was used (**Figure 7A**) (15). The levels of the NLRP3 inflammasome components IL-1β and active caspase-1 in the peritoneal fluids were significantly increased in MSU intraperitoneally injected mice, and these effects were reduced by oral administration of CS (30 mg/kg/day; total three times before MSU injection) and intraperitoneal injection of colchicine (1 mg/kg; one time before MSU injection), a medication used to treat gout (**Figure 7B**). The levels of NLRP3 and ASC in the peritoneal cells of MSU-injected mice were also reduced by CS and colchicine (**Figure 7C**). These results indicated that oral administration of CS inhibited the NLRP3 inflammasome in vivo. In addition, CS and colchicine also reduced the levels of the NLRP3 inflammasome-independent cytokines IL-6 and MCP-1 in the peritoneal fluids of MSU-injected mice (**Figure 7D**). Furthermore, peritoneal neutrophil influx was significantly increased in MSU-injected mice, and this effect was reduced by CS and colchicine (**Figure 7E**). These findings indicated that CS inhibits gouty inflammation in a mouse model.

## Discussion

The drug repositioning approach has attracted particular attention from researchers and the pharmaceutical industry because it is an effective, low-cost and riskless strategy to find new indications for existing drugs compared to traditional *de novo* drug development strategies (27). The NLRP3 inflammasome has attracted attention from researchers and the pharmaceutical industry, as it participates in many inflammatory and neurodegenerative diseases (2). The development of NLRP3 inflammasome inhibitors has become a promising strategy to combat diseases. Although an increasing number of small molecular NLRP3 inhibitors are in clinical trials or in development, no NLRP3 inhibitor has yet hit the market. In addition to the tool compound MCC950, another NLRP3-specific inhibitor, CY-09, a C172 analogue without cystic fibrosis transmembrane conductance regulator channel inhibitory activity, targets the NLRP3 ATP-binding motif, inhibiting NLRP3 ATPase activity and NLRP3 inflammasome activation. CY-09 shows a promising pharmacokinetic profile and exhibits therapeutic properties in mouse models of cryopyrin-associated autoinflammatory syndrome and type 2 diabetes (28).

Several existing old drugs have been demonstrated to exert inhibitory effects against the NLRP3 inflammasome. Glyburide, a type 2 diabetes drug, was the first repositioned drug identified to inhibit the NLRP3 inflammasome by blocking ATP-sensitive K^+^ channels and reducing LPS-induced lethality in mice (29). Tranilast is a clinically used drug that can improve allergic conjunctivitis, bronchial asthma, atypical dermatitis and hypertrophic scars (30). It has been demonstrated that tranilast directly binds to NLRP3 and inhibits NLRP3 oligomerization, which leads to the inhibition of the NLRP3 inflammasome in macrophages. Tranilast ameliorates NLRP3 inflammasome-associated diseases in mice, including gouty arthritis, cryopyrin-associated autoinflammatory syndromes, and type 2 diabetes (31). Colchicine is an old drug for the prevention and treatment of gout flares. An increasing number of studies have shown the potential benefits of colchicine on COVID-19 (32) and cardiovascular disease (33). As the NLRP3 inflammasome plays important roles in the pathogenesis of COVID-19 and cardiovascular disease (34, 35), colchicine was tested for its potential inhibitory activity towards the NLRP3 inflammasome. Recent studies have shown that colchicine inhibits the NLRP3 inflammasome by reducing pore formation induced by P_2_X_7_ (36), inhibiting caspase-1 activation (37), and suppressing microtubule synthesis (38). Daniels et al. demonstrated that the nonsteroidal anti-inflammatory drugs flufenamic acid, meclofenamic acid and mefenamic acid inhibited the NLRP3 inflammasome by inhibiting volume-regulated anion channels in macrophages, and mefenamic acid ameliorated Alzheimer’s disease in a mouse model (39). In addition, methylergometrine, a smooth muscle constrictor used in postpartum haemorrhage, has been repositioned as a potential NLRP3 inflammasome inhibitor (40).

CS has been demonstrated to exert anti-inflammatory activities *in vitro* and *in vivo*. CS significantly reduced the expression levels of IL-6 and transforming growth factor-β (TGF-β) in TNF-α-stimulated human embryonic kidney epithelial cells (41). CS decreased the expression of Th1 and Th2 cytokines in the lung tissues of mice with ovalbumin-induced allergic asthma (42). CS also exerts anti-inflammatory activity in microglia, as it reduced proinflammatory mediators (IL-6, IL-12p70, TNF-α, nitric oxide), increased anti-inflammatory cytokines (IL-10 and TGF-β) and shifted microglia to the anti-inflammatory M2 phenotype in LPS + interferon-γ-activated microglia (43). As CS showed an antineuroinflammatory effect *in vitro*, CS significantly reduced the amyloid burden and microglial activation in the hippocampus of an Alzheimer’s disease mouse model (44) and has been used in clinical trials for Alzheimer’s disease (45). Importantly, CS has the potential to ameliorate the COVID-19 cytokine storm (46). It has been demonstrated that the NLRP3 inflammasome is crucial for COVID-19 and the development of Alzheimer’s disease (34, 47). We suggest that CS ameliorates COVID-19 and Alzheimer’s disease, at least in part, by inhibiting the NLRP3 inflammasome.

Notably, although CS inhibited mitochondrial ROS production, it cannot explain the mode of action of CS-mediated inhibition of the NLRP3 inflammasome. We found that CS inhibited the activation of the caspase-11 (**Figure 2A**) and GSDMD (**Figure 2B**) in nigericin-activated J774A.1 macrophages. CS also reduced cell death (**Figure 2C**) and release of LDH (**Figure 2D**), NLRP3 and ASC (**Figure 2E**) in nigericin-activated J774A.1 macrophages. These results indicate that CS significantly inhibited pyroptosis and membrane permeability induced by nigericin. Taken together, we suggest that CS inhibited the NLRP3 inflammasome and reduced IL-1β and IL-18 secretion through reduced mitochondrial ROS inhibition and pyroptosis. In addition, CS reduced the expression levels of TNF-α and IL-6 by reducing ROS generation and NF-κB activation in LPS-activated human monocytes. CS also reduced LPS-induced mRNA expression of IL-1β (48). In this study we found that CS reduced proIL-1β and NLRP3 expression in LPS-activated macrophages, and these effects may be due to its inhibitory effects on the activation of NF-κB, JNK1/2 and ERK1/2 because we demonstrated that LPS induced NLRP3 through NF-κB, JNK1/2 and ERK1/2 (20). As CS inhibited the phosphorylation levels of ERK1/2 and JNK1/2 (**Figure 3D**) as well as inhibited NF-κB activation (**Figures 3E, F**) in LPS-activated macrophages, we suggested that CS not only inhibited the NLRP3 inflammasome, but also had broad anti-inflammatory effects in macrophages. Although CS reduced ROS generation in LPS-stimulated monocytes (48), we found that CS did not reduce but increased ROS generation in LPS-stimulated macrophages. The opposite effect of CS on ROS generation may be cell type dependent. In general, ROS promotes NLRP3 inflammasome activation (20); however, one interesting study from Erttmann and Gekara found an unexpected role of hydrogen peroxide on NLRP3 inflammasome activation. Erttmann and Gekara demonstrated that hydrogen peroxide released by *Streptococcus pneumoniae* inhibited the NLRP3 inflammasome (49). However, the role of increased hydrogen peroxide levels in CS-treated macrophages on the NLRP3 inflammasome requires further investigation.

It should be noted that CS significantly inhibited caspase-1 activation at 15 µM, but significantly reduced IL-1β and IL-18 secretion at 7.5 µM in nigericin-activated J774A.1 macrophages. One of the explanations to this difference is CS at 7.5 µM significantly inhibited caspase-11 activation (**Figure 2A**), GSDMD cleavage (**Figure 2B**) and LDH release (**Figure 2D**) in nigericin-activated J774A.1 macrophages. These results indicate that nigericin-induced membrane permeability increased (pyroptosis) were reduced by candesartan, which may explain why CS at 7.5 µM reduced secretion of IL-1β and IL-18. In addition, although CS reduced the expression levels of ASC in the supernatants of nigericin-activated (**Figure 2E**) and ATP-activated (**Figure 2F**) J774A.1 macrophages, ASC constitute expressed in the J774A.1 macrophages and it is not affected by LPS, nigericin or candesartan (**Figure 5D**). We suggest that the reduced ASC expression in the supernatants by candesartan is mainly resulted from the reduced membrane permeability and pyroptosis, but not reducing the ASC protein expression levels.

It is important to understand whether the NLRP3 inflammasome inhibitory effect of CS is mediated *via* angiotensin II receptor type 1 inhibition and blood pressure lowering activity or through the off-target effect. Although we demonstrated that another angiotensin II receptor blocker irbesartan inhibited IL-1β secretion in nigericin-activated J774A.1 macrophages (**Figure 1J**), we cannot rule out the possibility that CS inhibited the NLRP3 inflammasome through angiotensin II receptor type 1 independent pathways. The limitation of this study is not testing the effect of knockout or knockdown of angiotensin II receptor type 1 on the NLRP3 inflammasome activation. In the previous study, CS significantly suppressed TGF-β and IL-6 expression in TNF-α-stimulated human embryonic kidney epithelial cells. Surprisingly, silence of angiotensin II type 1 receptor by siRNA did not affect CS-mediated inhibition of TGF-β and IL-6 expression. These results indicated that CS inhibits inflammation through an angiotensin II type 1 receptor independent pathways (41). In addition, the anti-inflammatory effects of CS on LPS-activated human monocytes were independent of its angiotensin II receptor type 1 inhibitory activity, as human monocytes did not express detectable angiotensin II receptor type 1 (48). Furthermore, spontaneously hypertensive rats that received high-dose CS (75 mg/kg per day) ameliorated renal injury by significantly reducing renal inflammation and macrophage infiltration. However, although low-dose CS (5 mg/kg per day) reduced blood pressure similar to high-dose CS, low-dose CS did not show a renal protective effect. These results indicated that the renal protective effect of CS was not dependent on its blood pressure lowering activity (50). Another limitation of this study is that we only investigated the effect of CS on MSU-induced peritonitis in a mouse model and no other *in vivo* models were tested. It is worthwhile to test the effect of CS on other NLRP3-associated disease models in the future, such as inflammatory bowel disease (51) and Huntington’s disease (52). Although there were no side effects observed in mice during this study, the potential side effects of CS should be monitored, especially in the models with long term CS treatment.

## Data Availability Statement

The original contributions presented in the study are included in the article/**Supplementary Material**. Further inquiries can be directed to the corresponding author.

## Ethics Statement

The studies involving human participants were reviewed and approved by Institutional Review Board of the Tri-Service General Hospital, National Defense Medical Center (TSGH-IRB-2-106-05-190 and TSGH-IRB-2-106-05-009). The patients/participants provided their written informed consent to participate in this study. The animal study was reviewed and approved by Institutional Animal Care and Use Committee of the National Ilan University.

## Author Contributions

K-FH is the guarantor of the article. W-YL and K-FH conceived and designed the study. W-YL, L-HL, Y-YH, W-TW, H-WC, and H-TH performed the experiments and analyzed the data. Y-JP, C-LH, and OC assisted with some experiments. S-MC and S-PY contributed to critical revision of the manuscript. W-YL, L-HL, and K-FH wrote and finished the manuscript. All authors participated in revising the manuscript and approved the final version.

## Funding

This research work is supported by the funding from the Ministry of Science and Technology of Taiwan (MOST 110-2628-B-197-001; MOST 110-2923-B-197-001-MY3; MOST 110-2811-B-197-002) and Tri-Service General Hospital, National Defense Medical Center, Taipei, Taiwan (TSGH-E-109208; 20170211).

## Conflict of Interest

The authors declare that the research was conducted in the absence of any commercial or financial relationships that could be construed as a potential conflict of interest.

## Publisher’s Note

All claims expressed in this article are solely those of the authors and do not necessarily represent those of their affiliated organizations, or those of the publisher, the editors and the reviewers. Any product that may be evaluated in this article, or claim that may be made by its manufacturer, is not guaranteed or endorsed by the publisher.
