# Supplementary figures and images for: Repositioning of the Angiotensin II Receptor Antagonist Candesartan as an Anti-Inflammatory Agent With NLRP3 Inflammasome Inhibitory Activity
Source: Front Immunol. 2022 May 20;13:870627. doi: 10.3389/fimmu.2022.870627 (PMC9163344; doi:10.3389/fimmu.2022.870627)

**A**

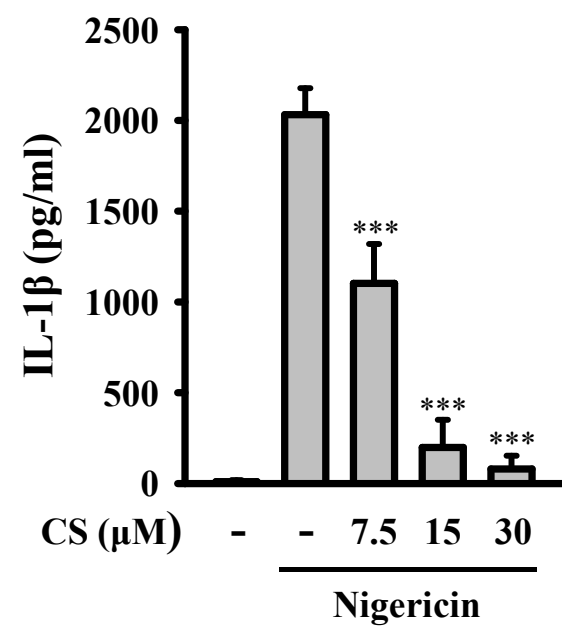

**B**

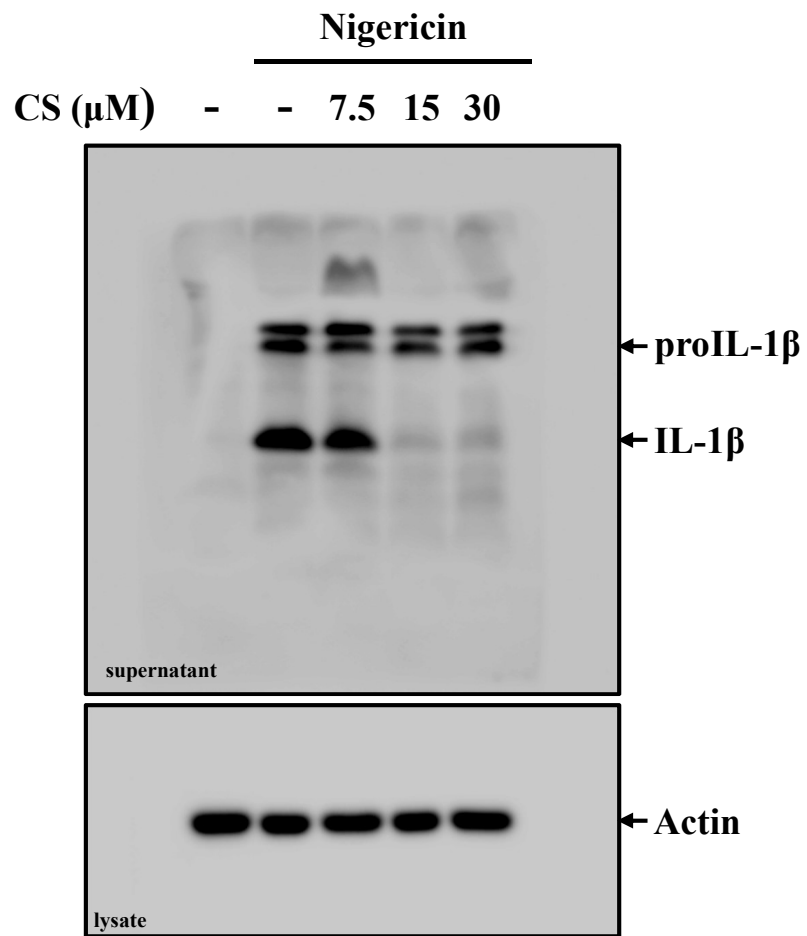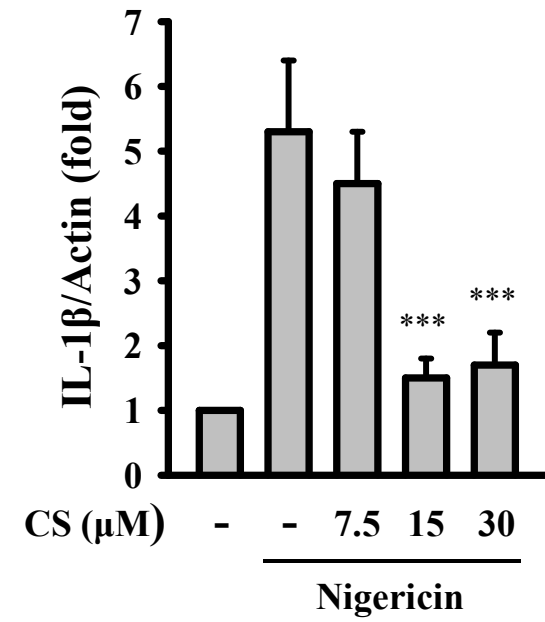

**C**

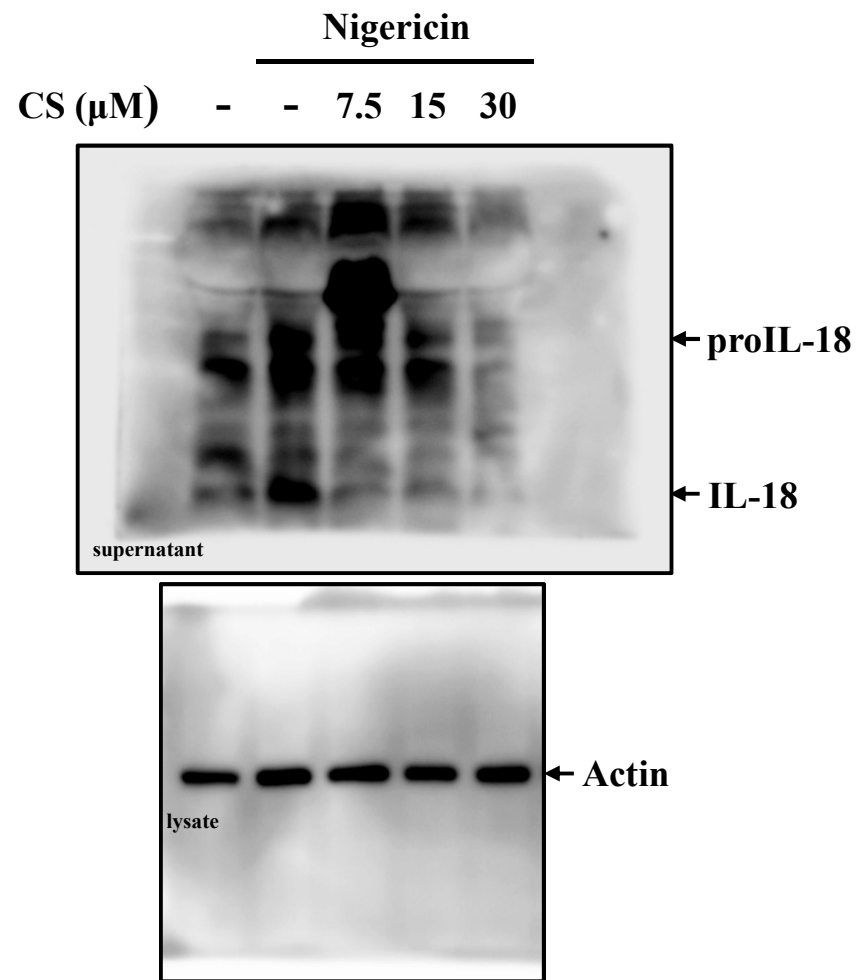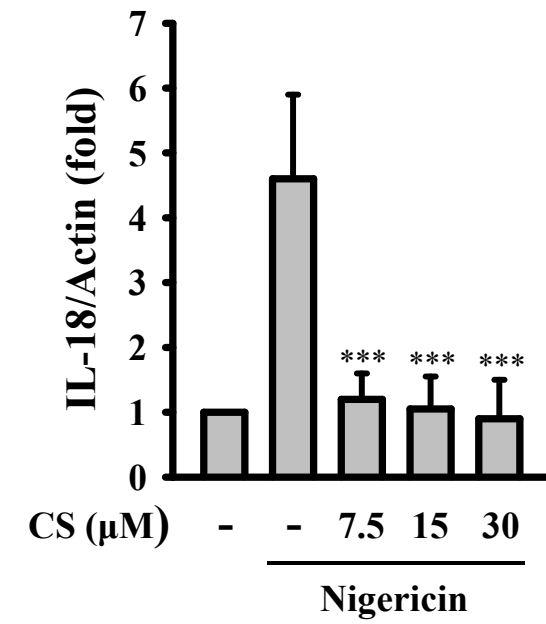

**D**

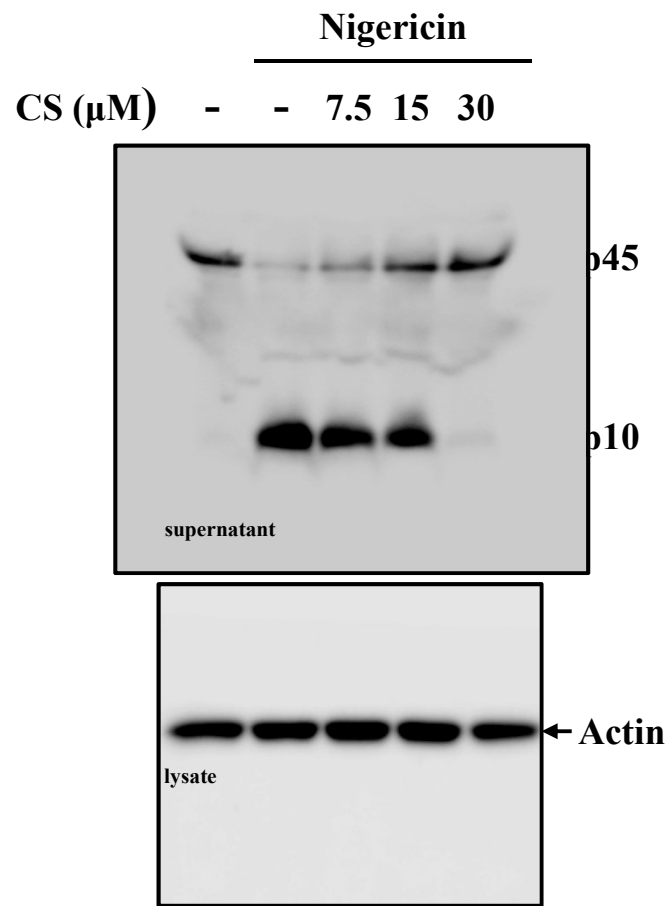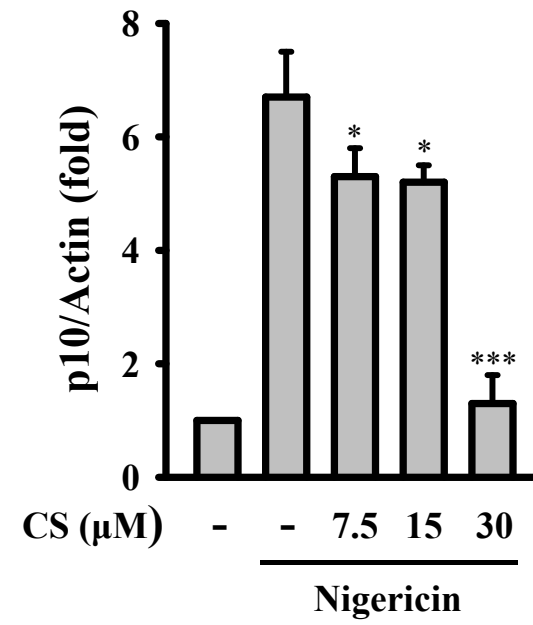

**E**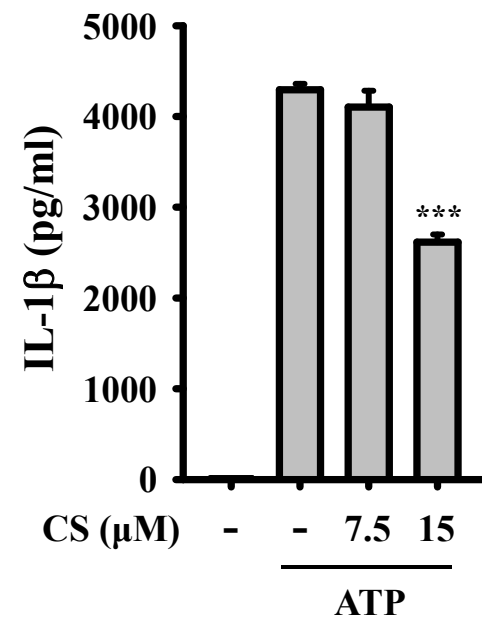**F**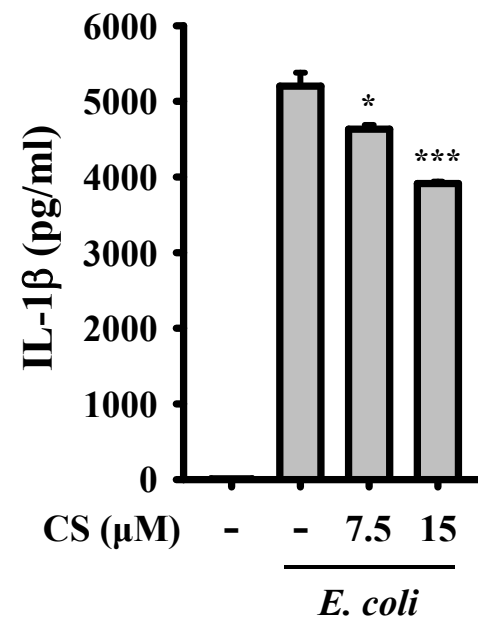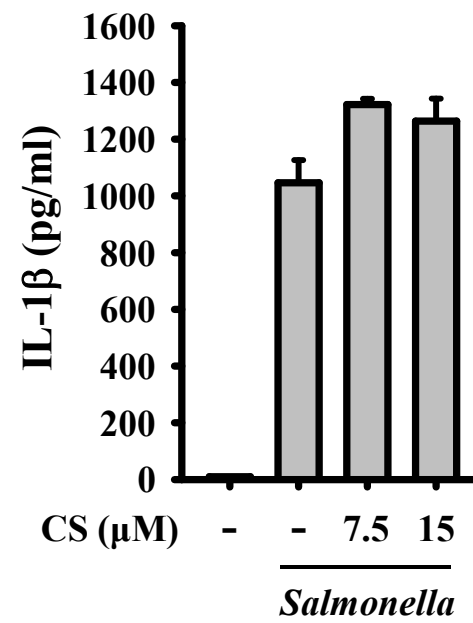

**A**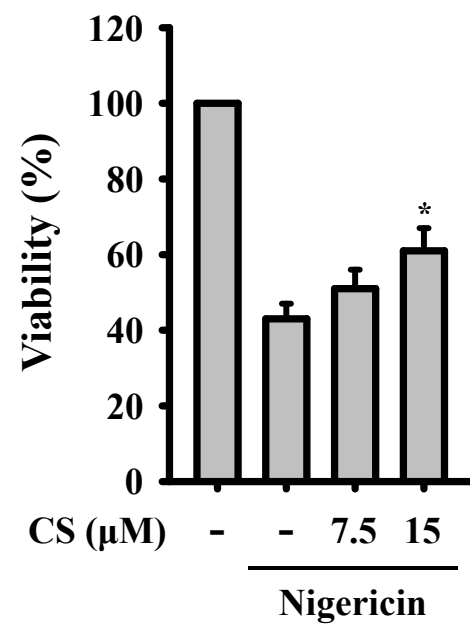**B**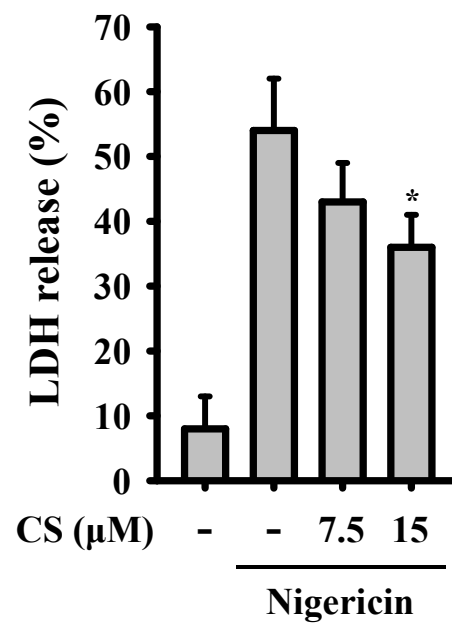

C

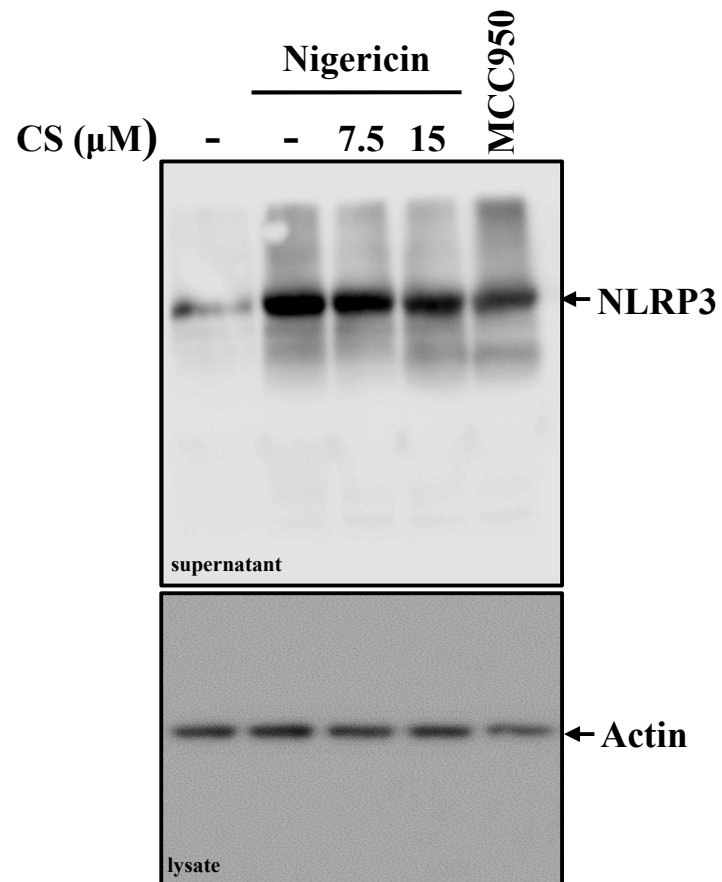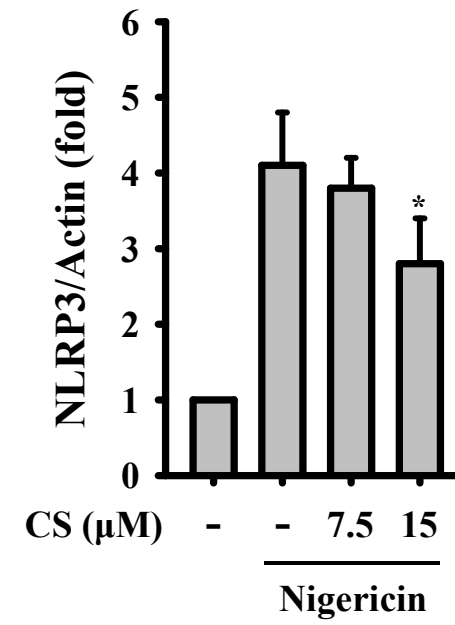

**D**

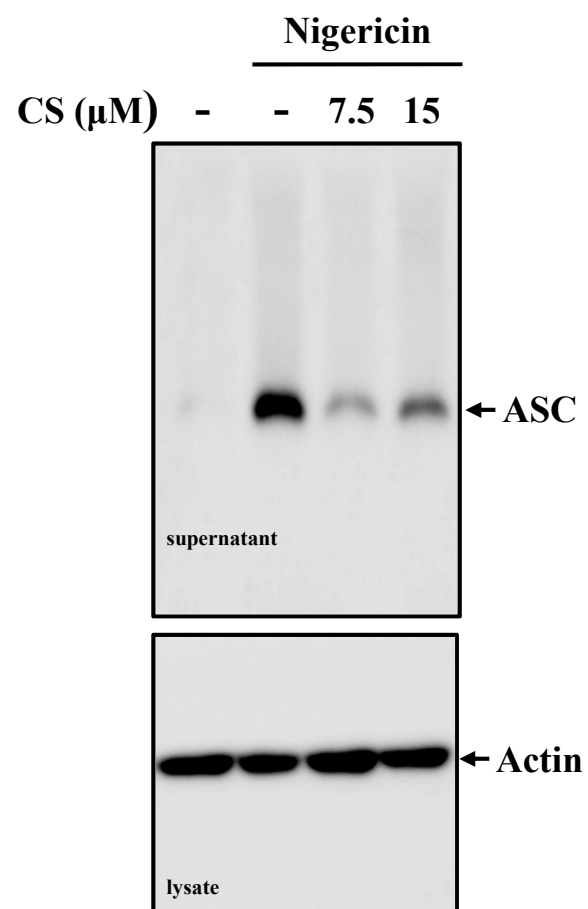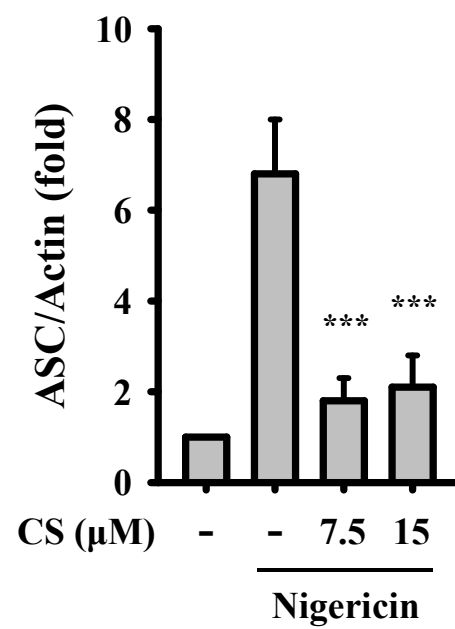

**A**

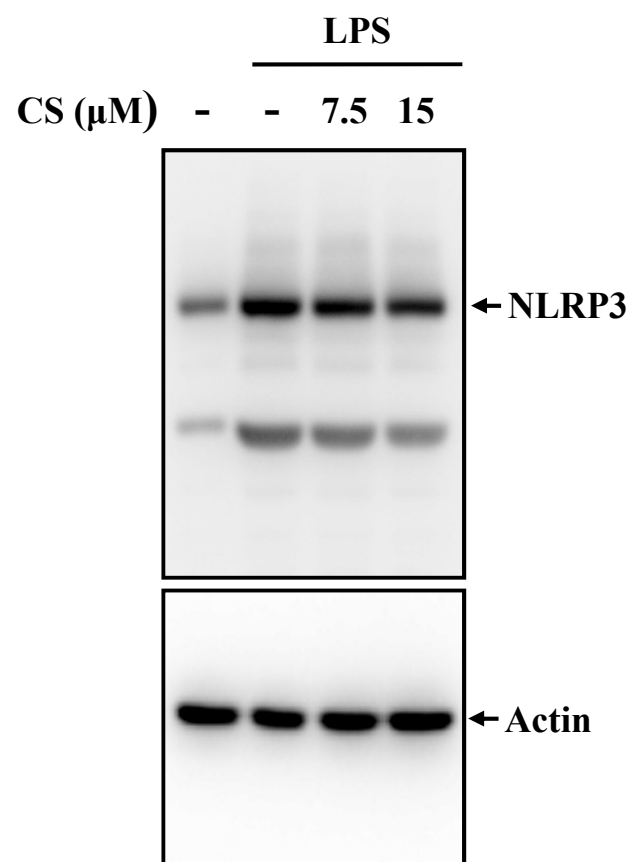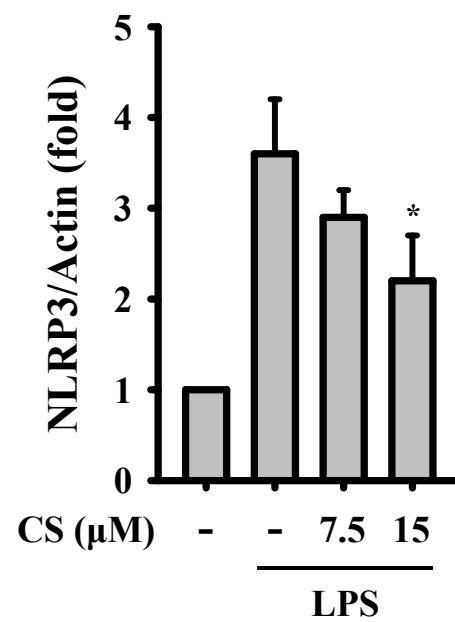

**B**

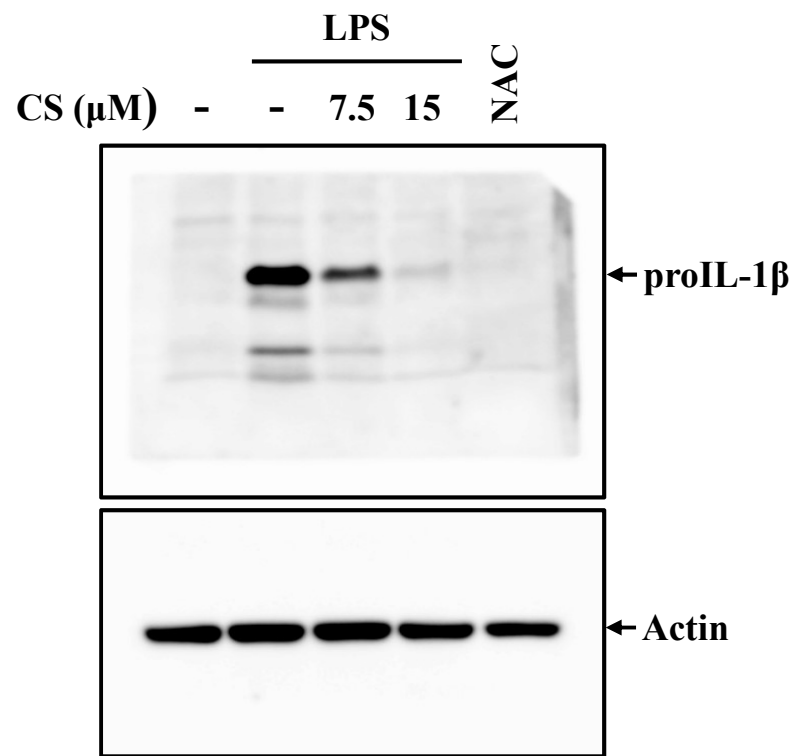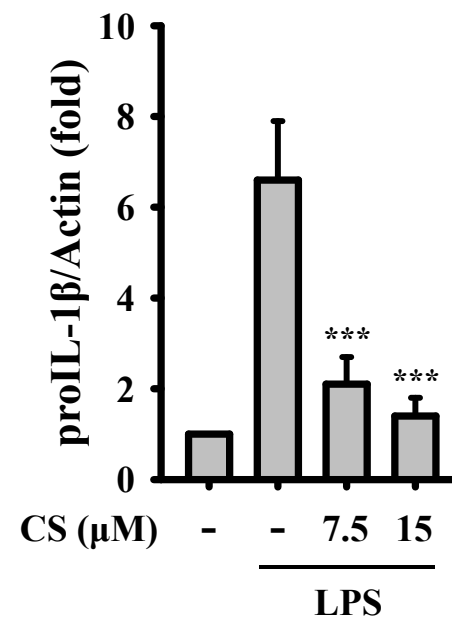

C

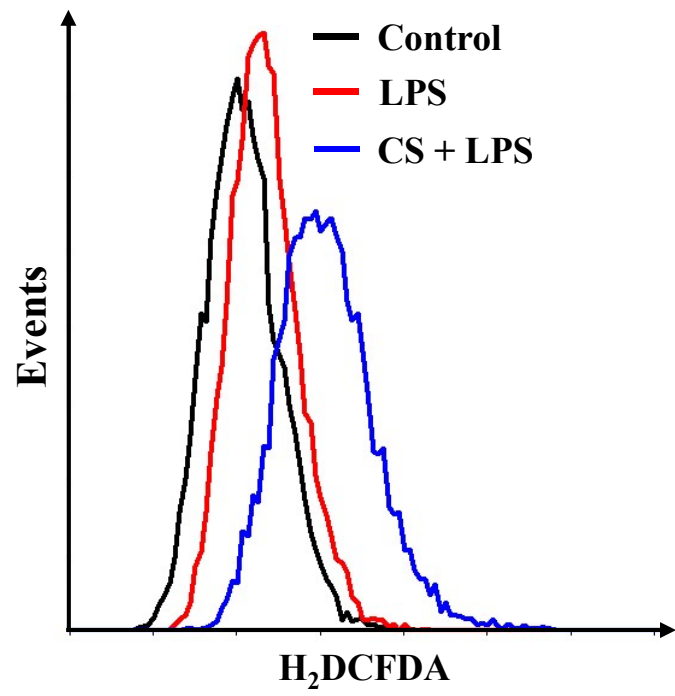

**D**

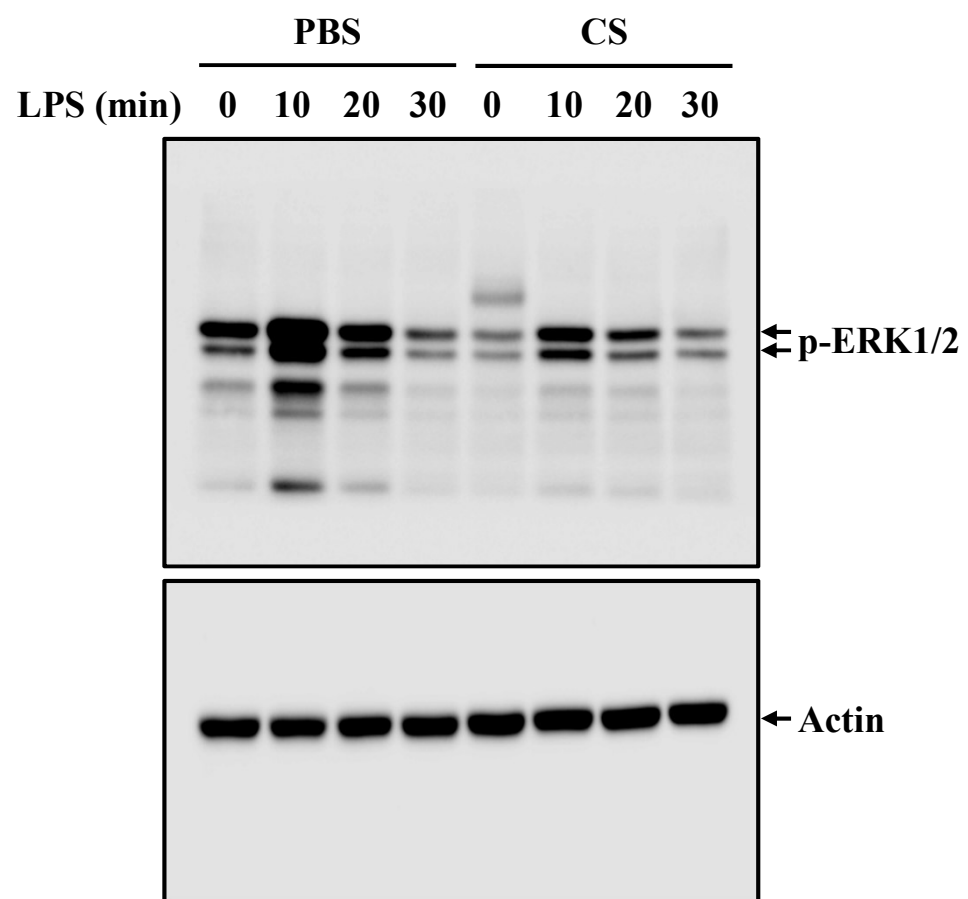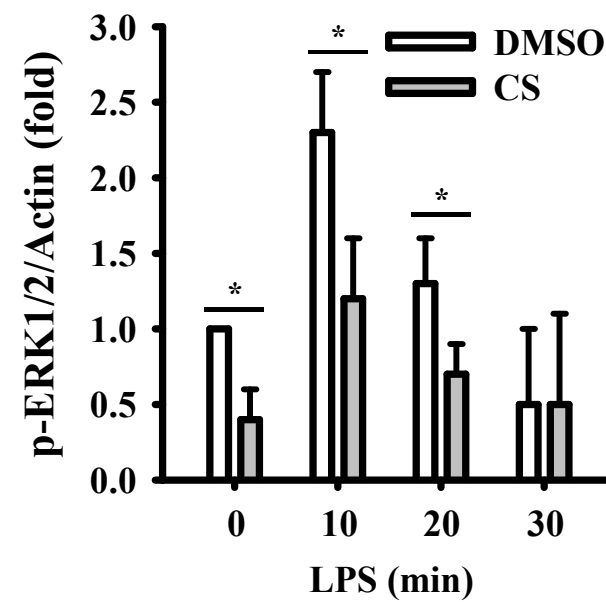

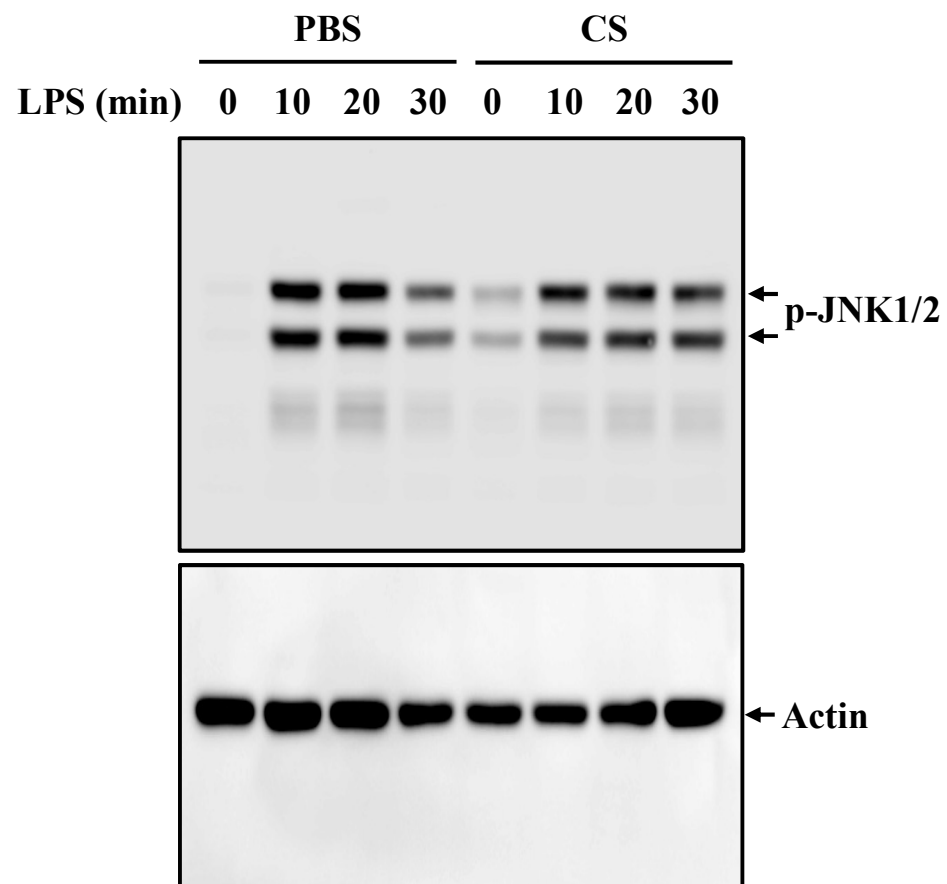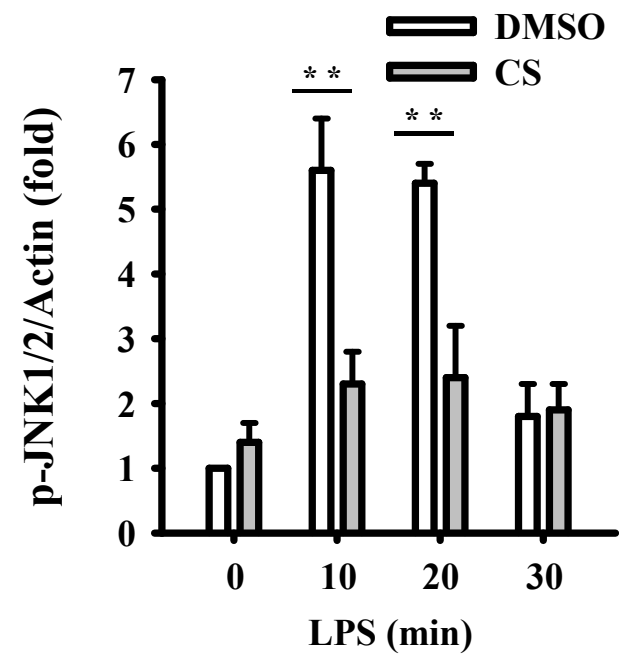

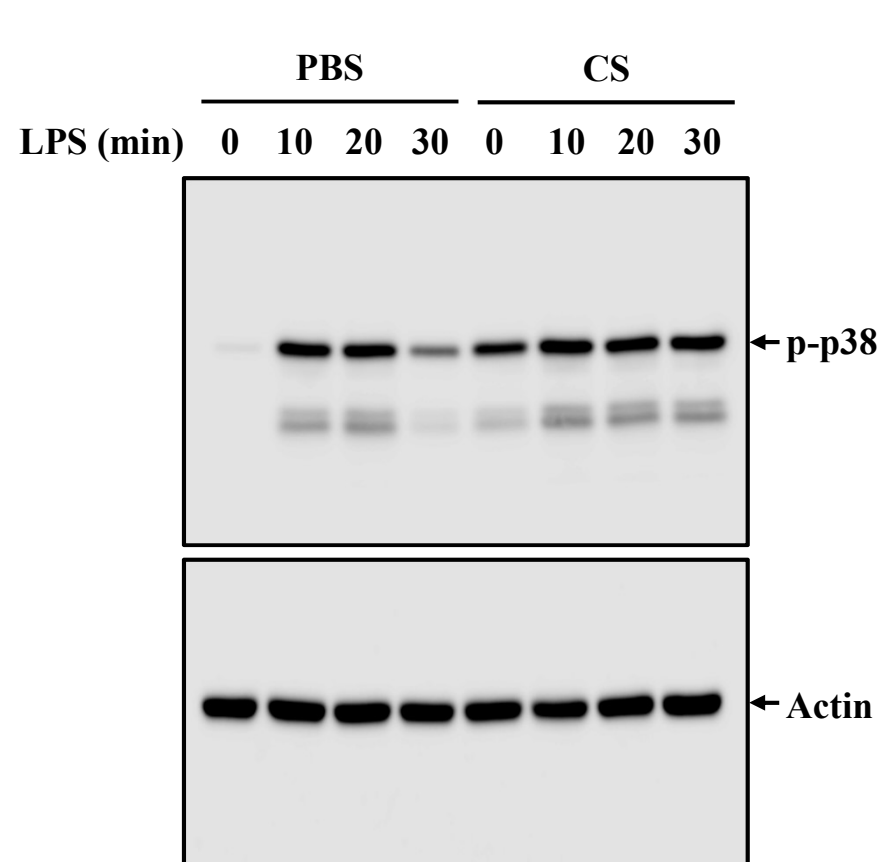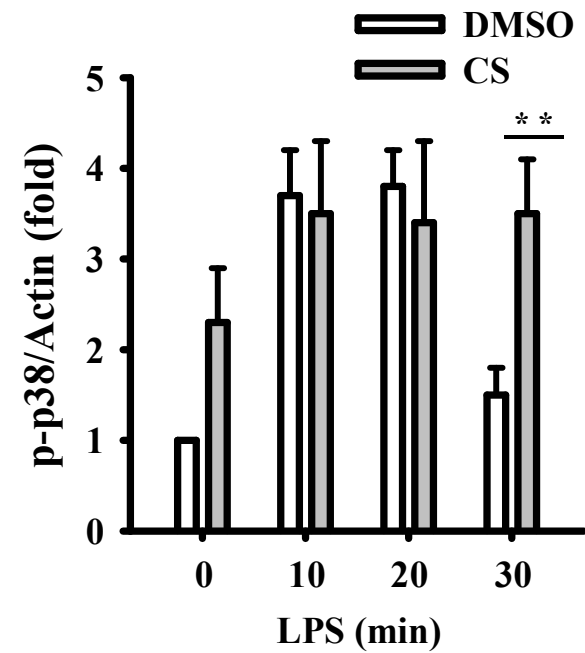

**E**

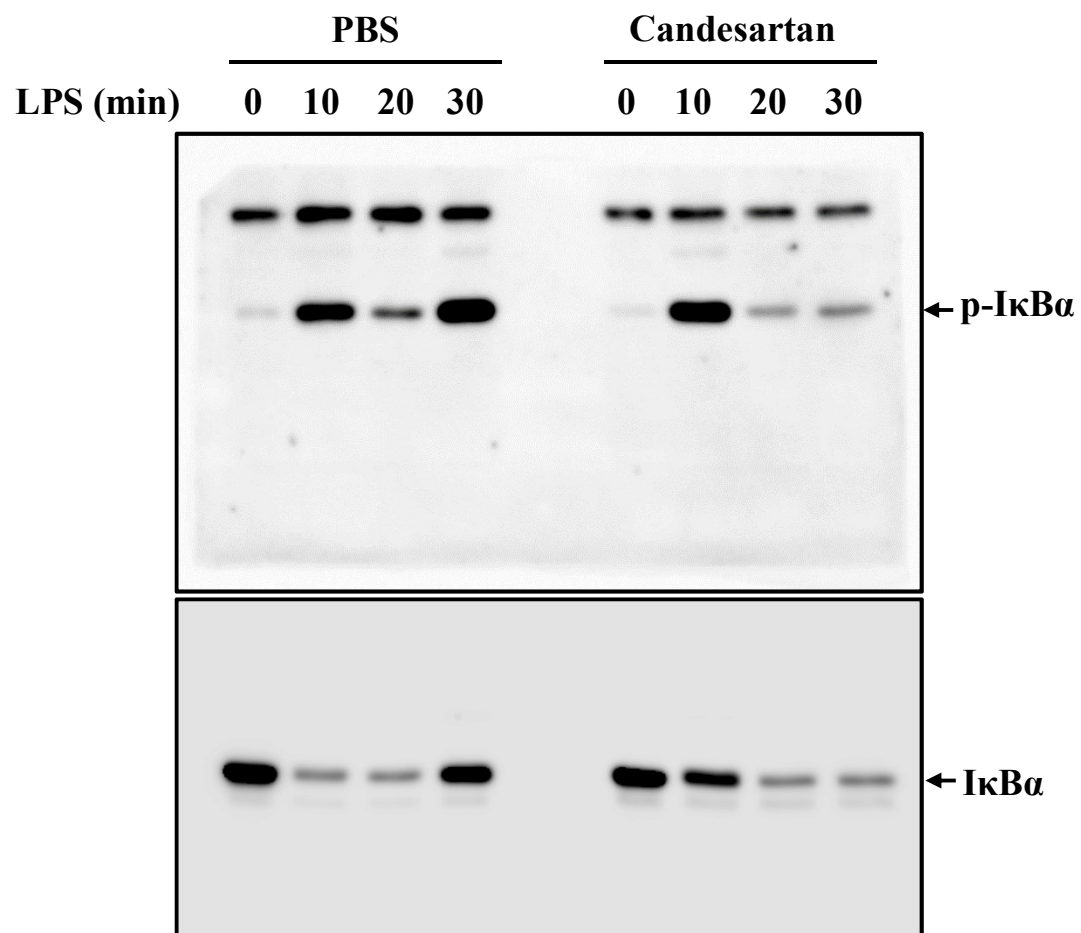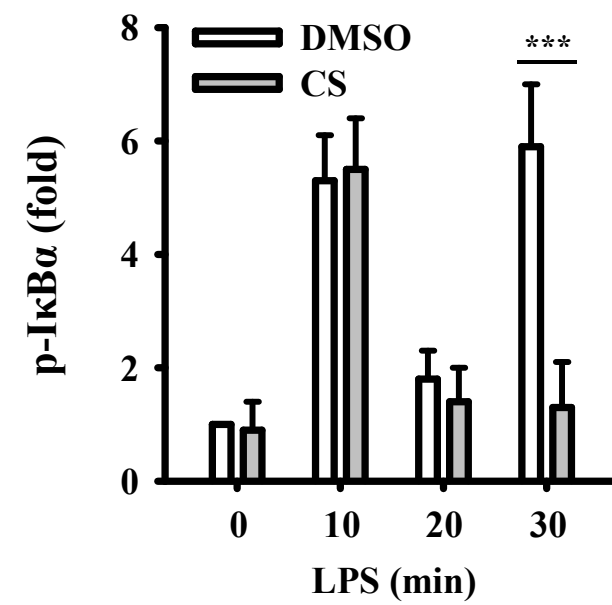

**F**

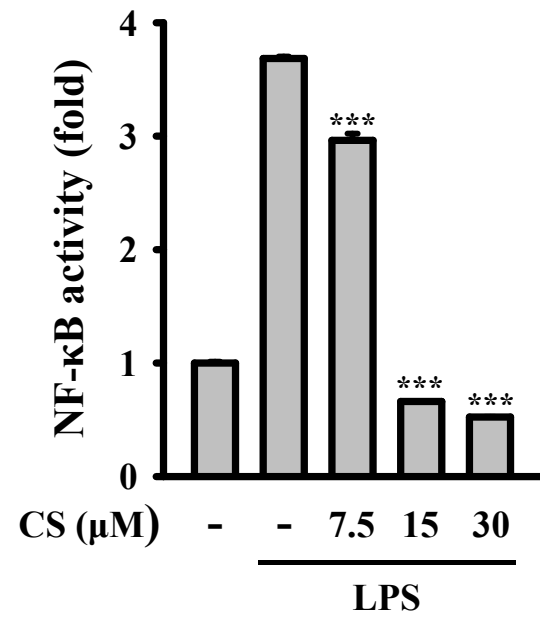

**A**

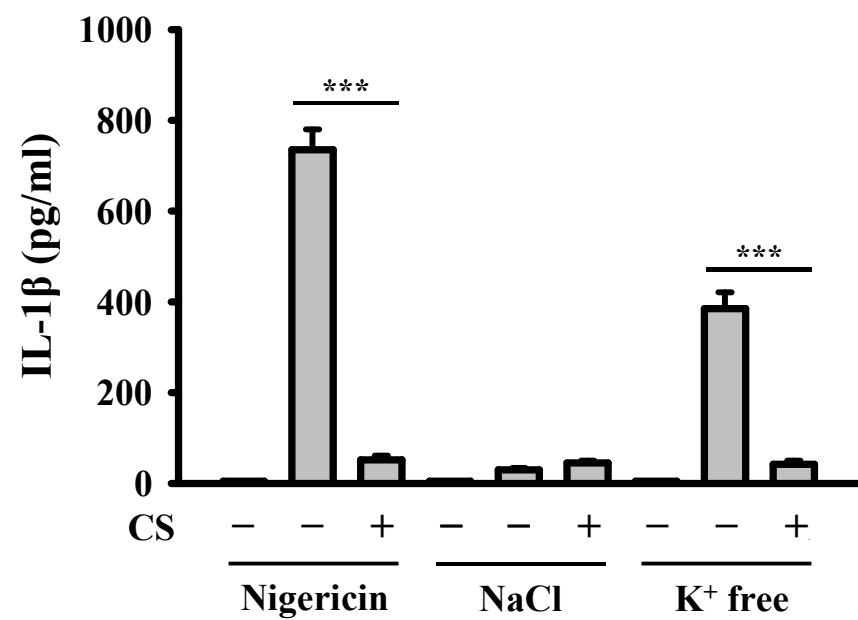

**B**

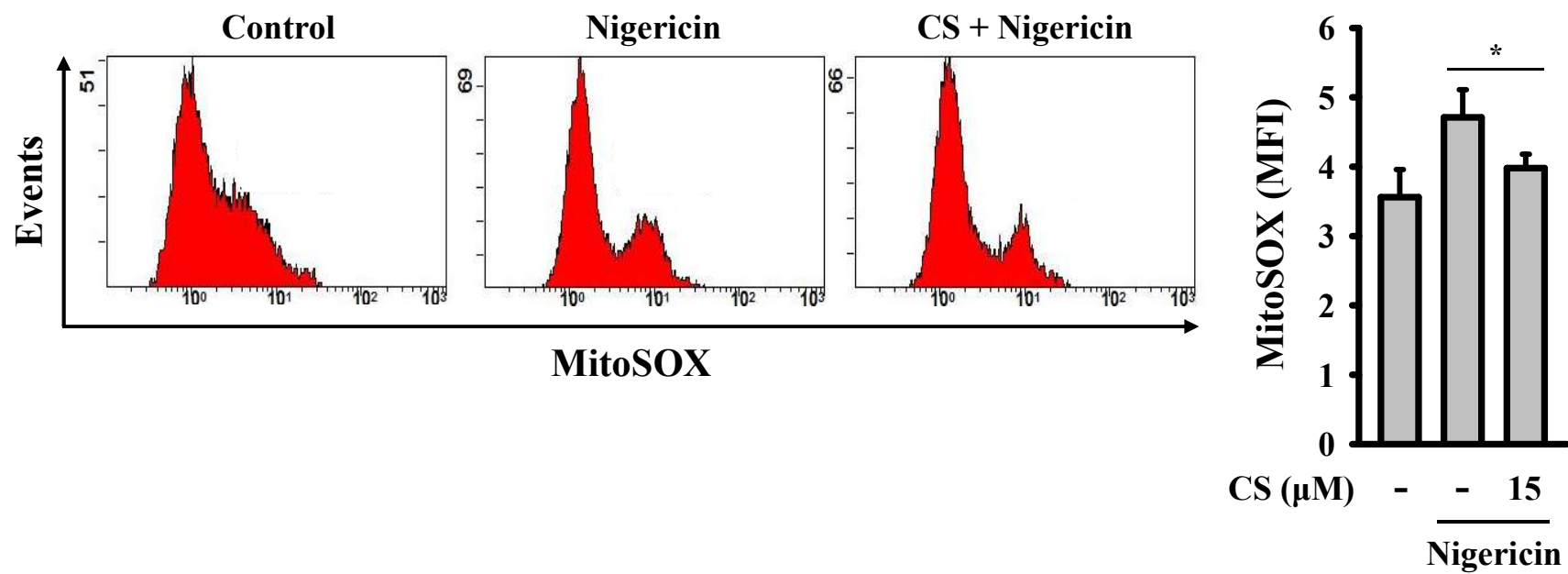

C

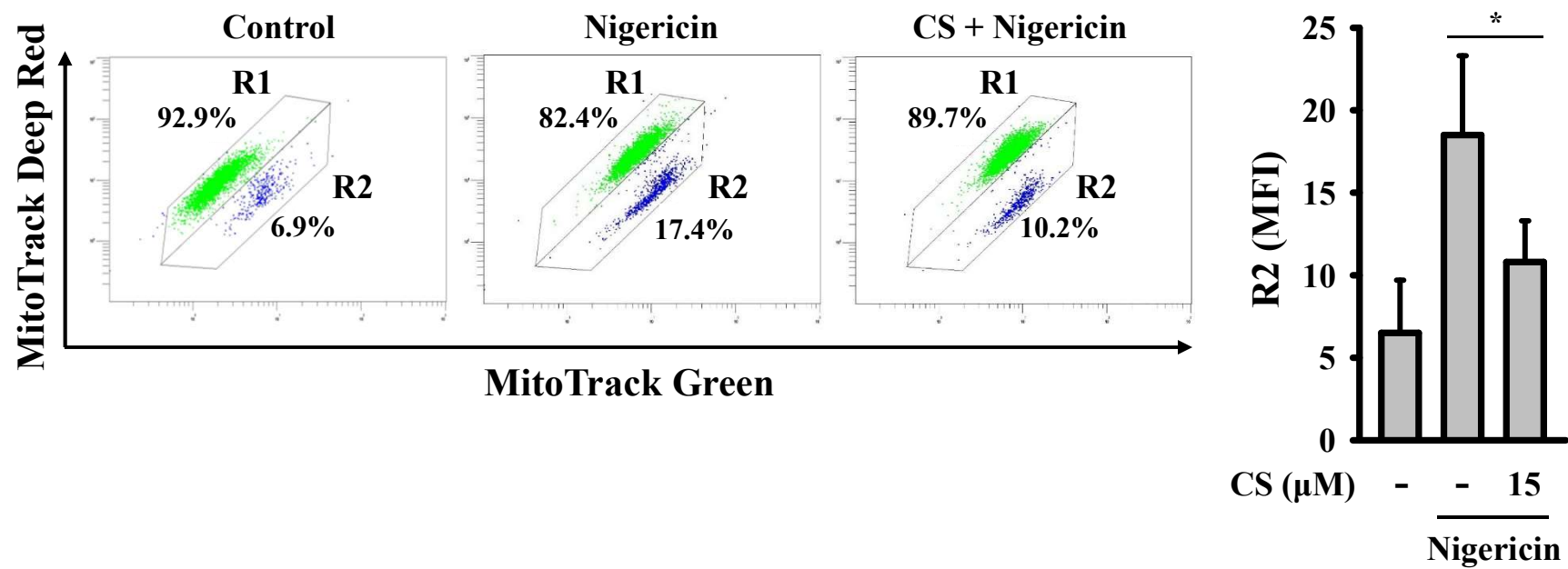

**D**

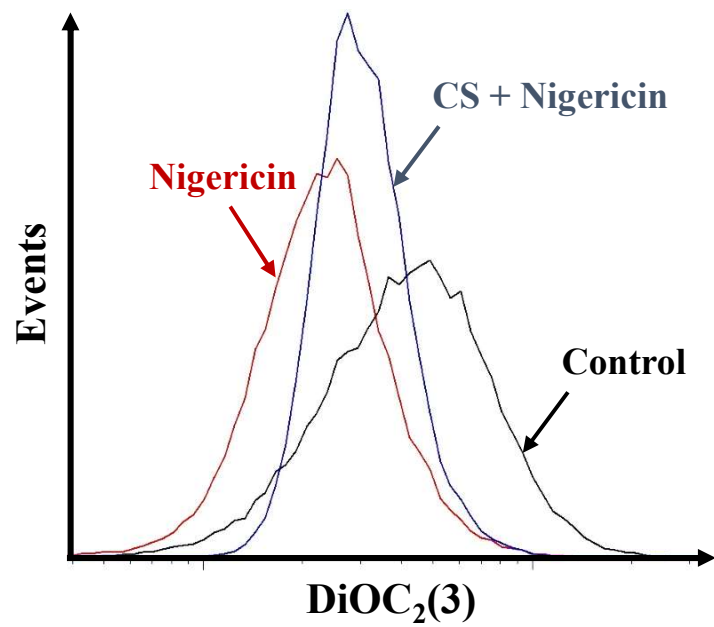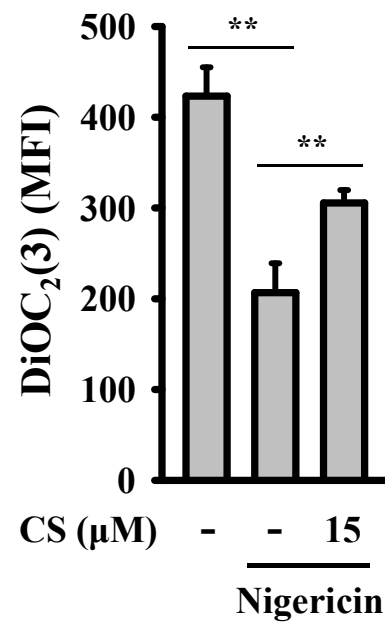

**A**

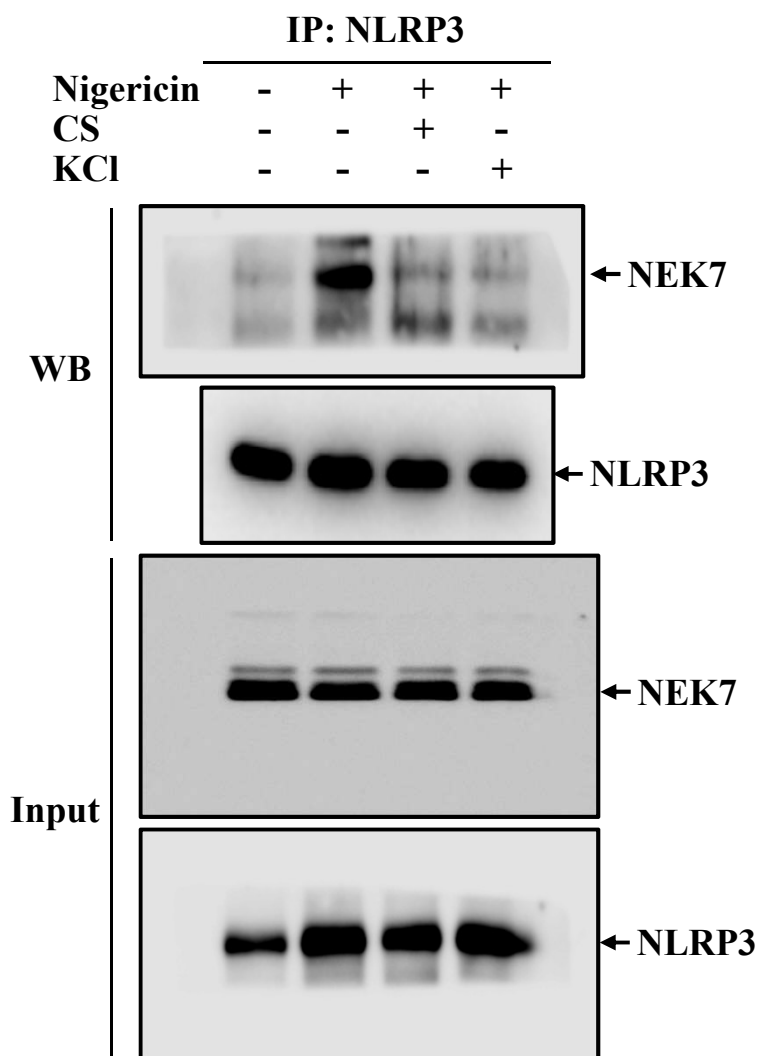

**B**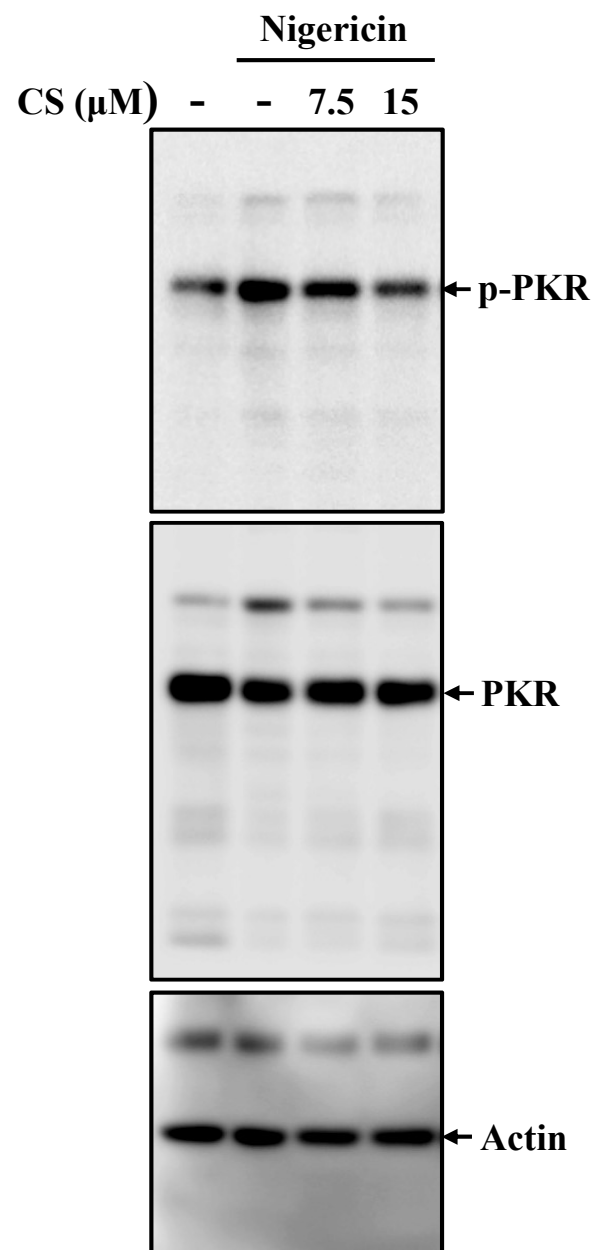**C**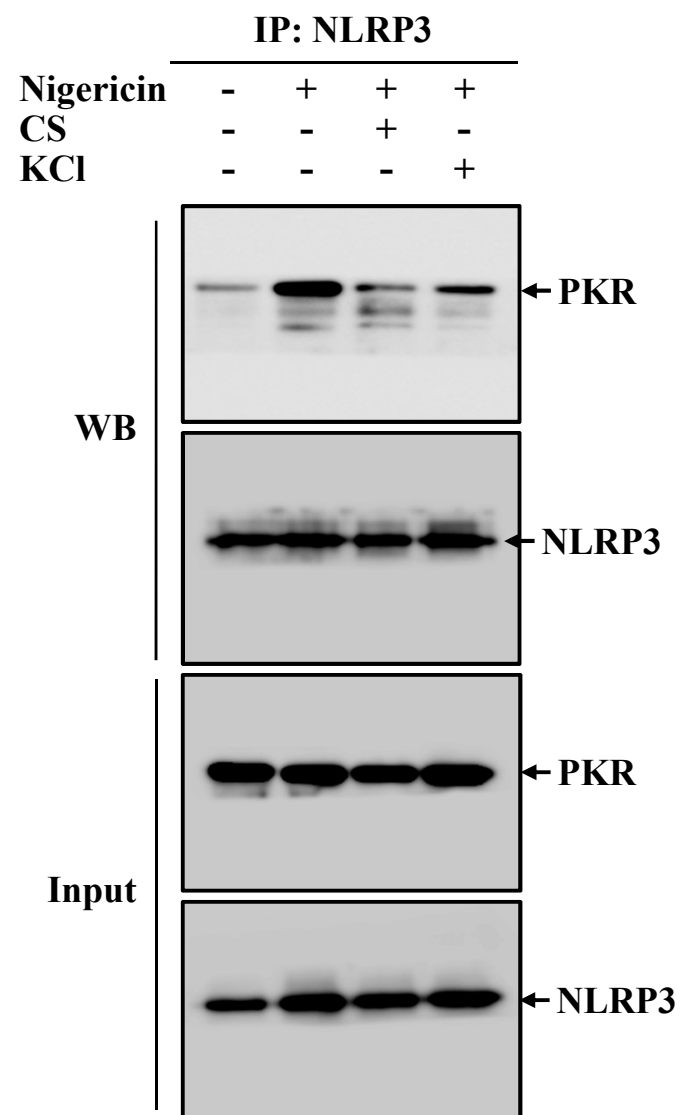

**D**

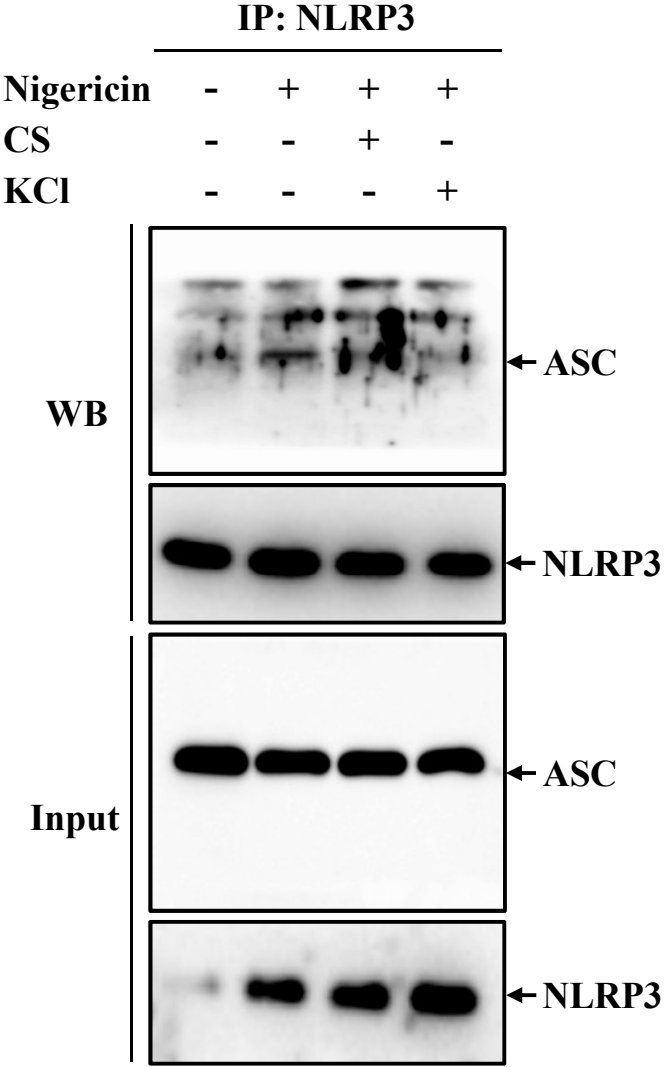

**A**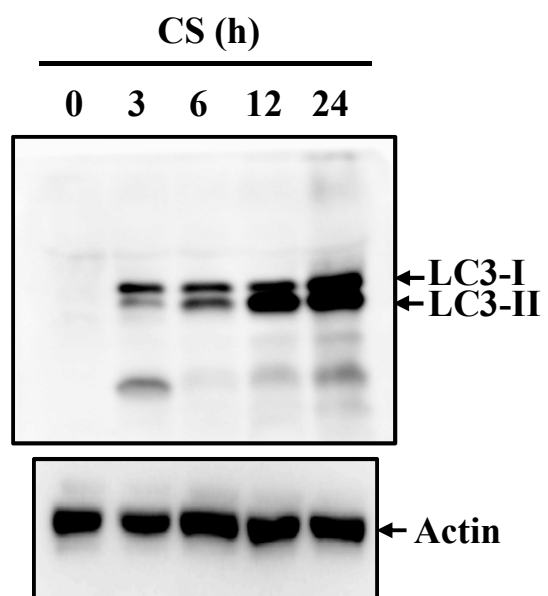**B**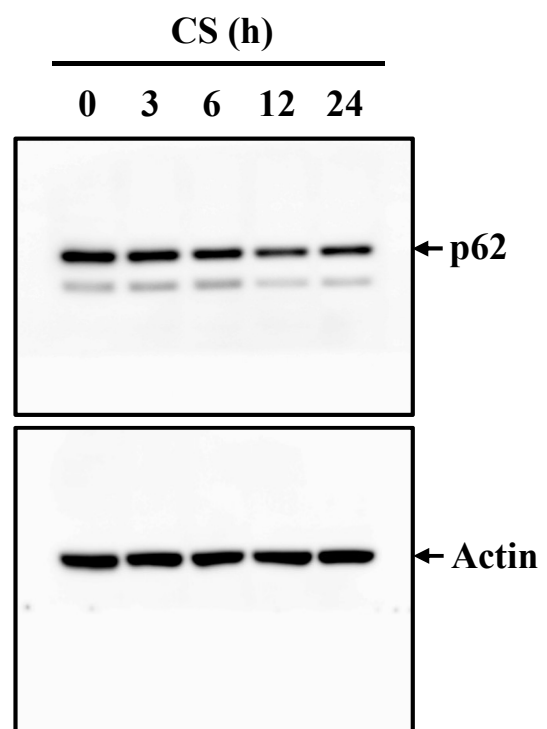

**C**

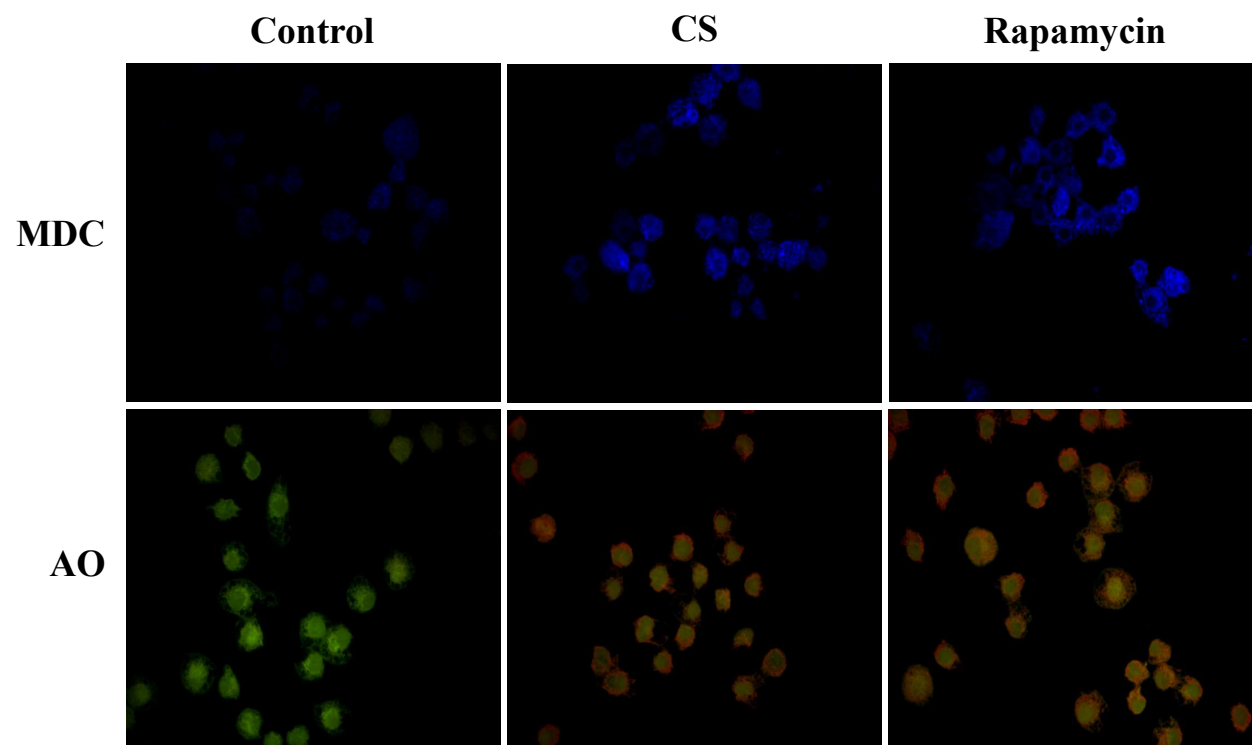

D

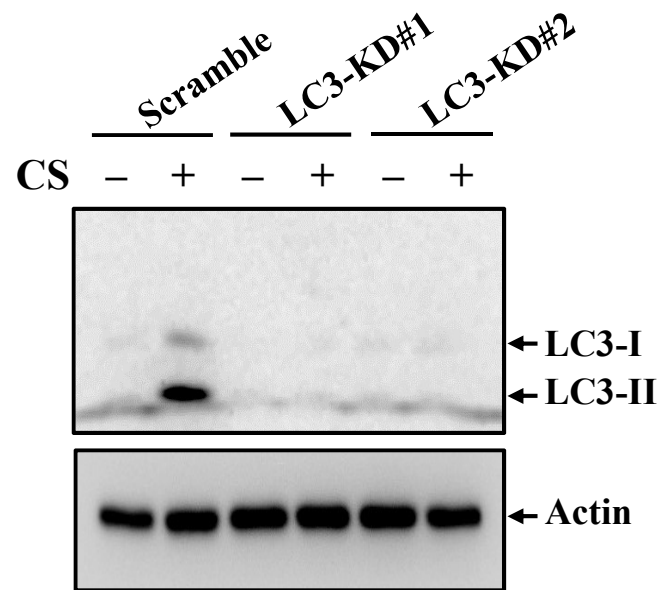

**E**

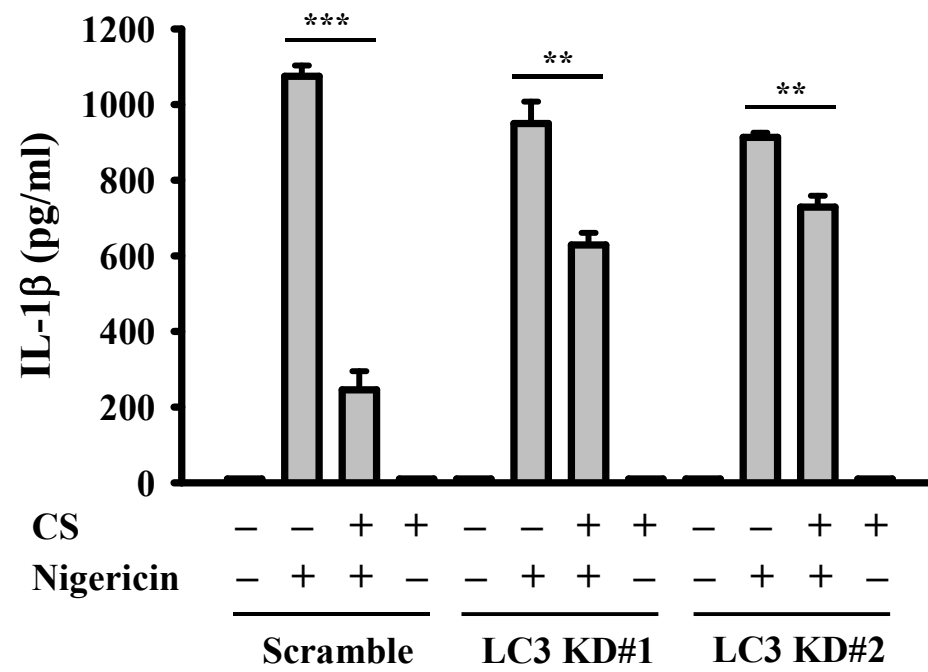

**A**

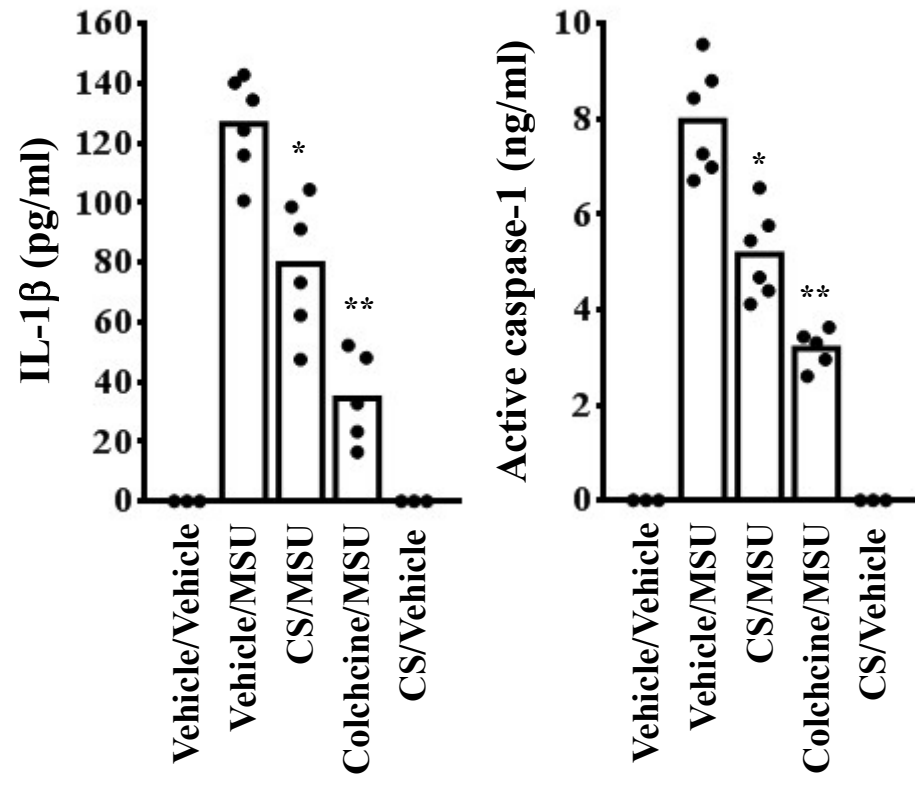

**B**

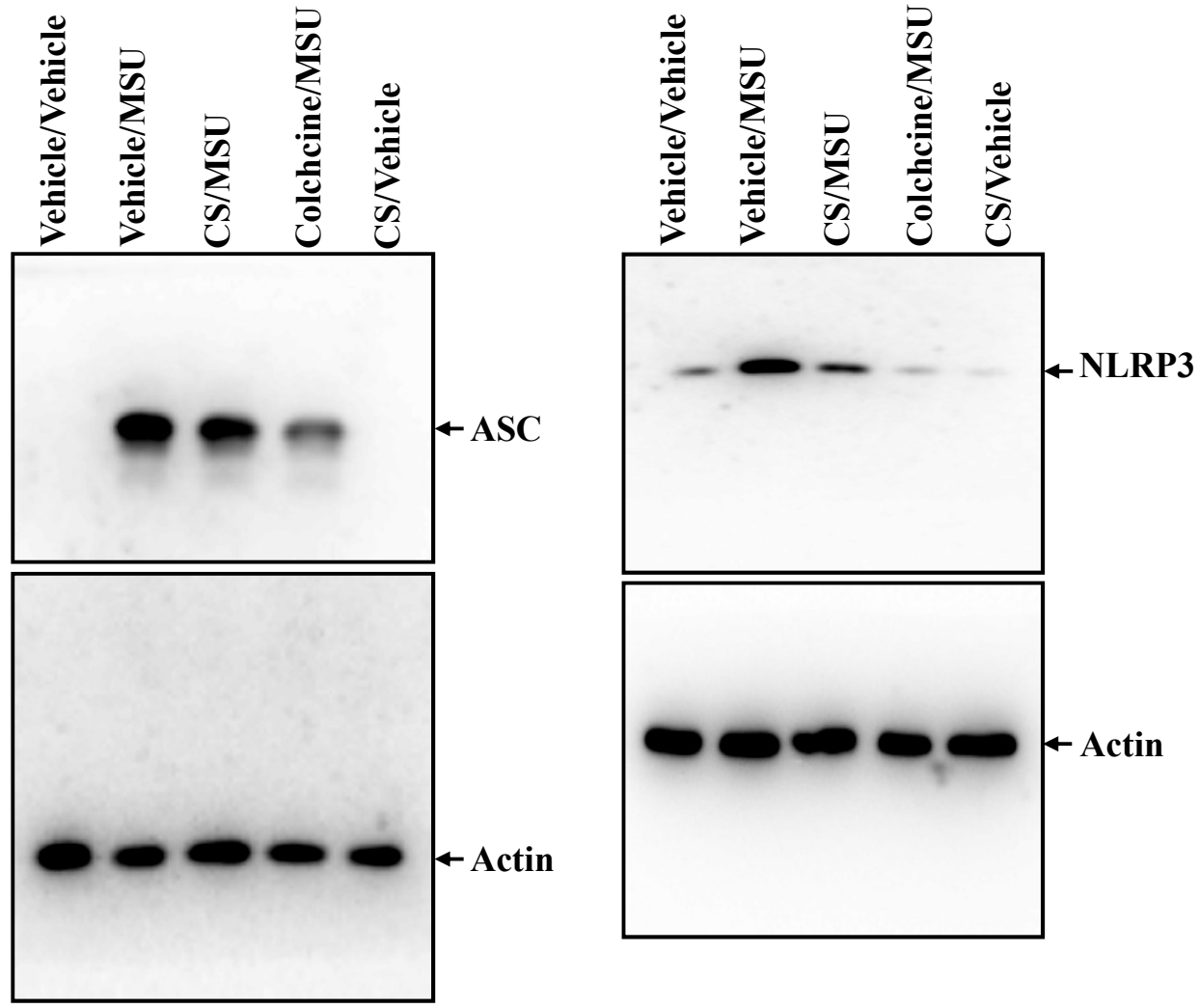

**C**

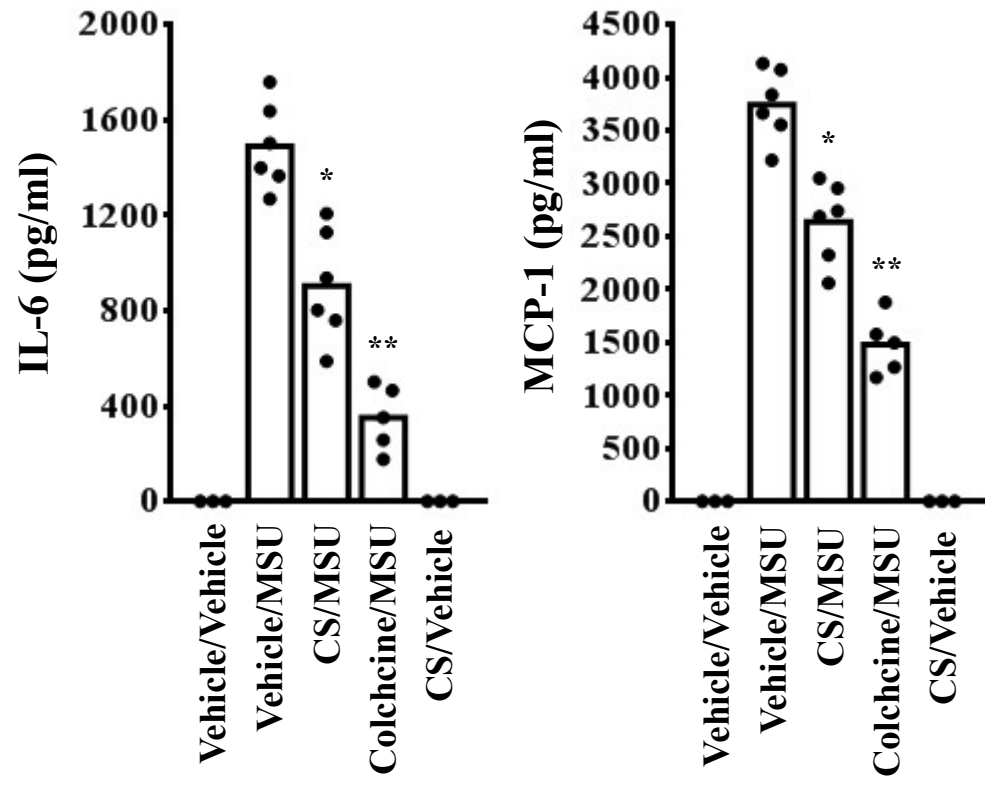

**D**

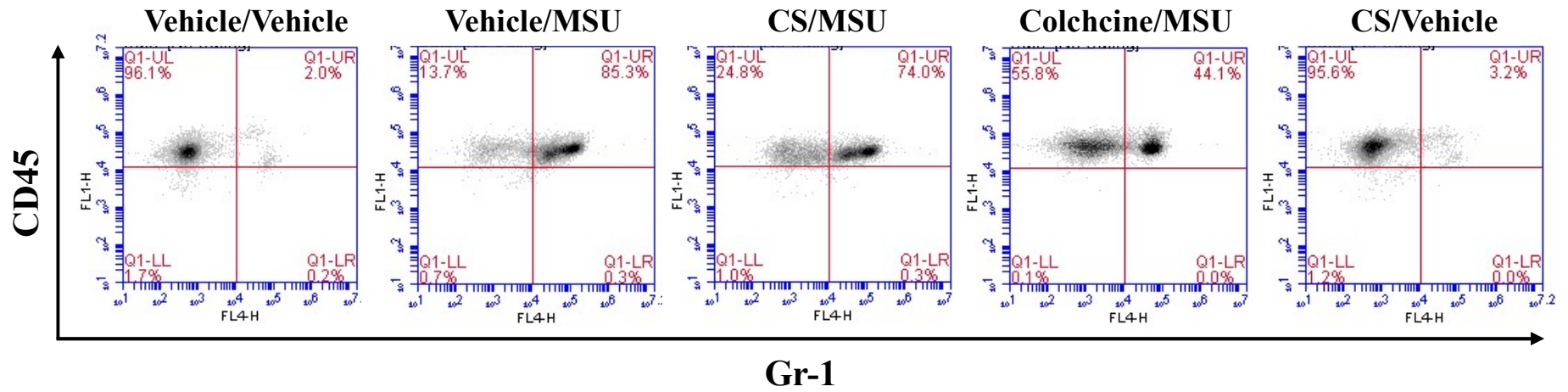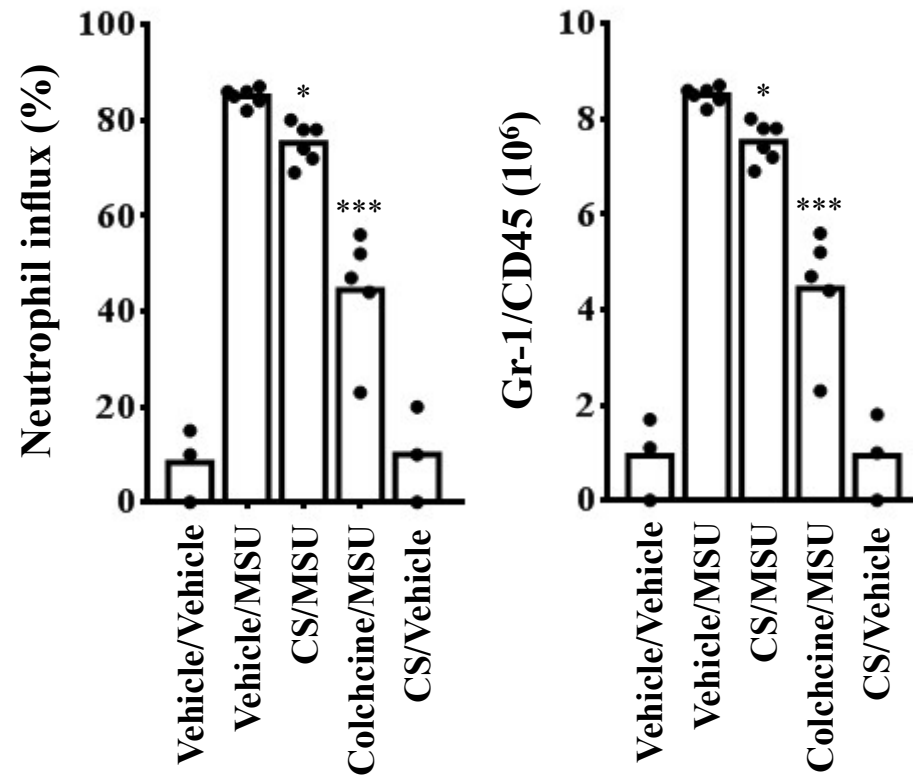

Supplement: Supplementary file 1 [file DataSheet_1.pdf]
